# Diabetic foot complications among Indigenous peoples in Canada: a scoping review through the *PROGRESS-PLUS* equity lens

**DOI:** 10.3389/fendo.2023.1177020

**Published:** 2023-08-14

**Authors:** Virginie Blanchette, Jérôme Patry, Magali Brousseau-Foley, Shweta Todkar, Solène Libier, Anne-Marie Leclerc, David G. Armstrong, Marie-Claude Tremblay

**Affiliations:** ^1^ Department of Human Kinetics and Podiatric Medicine, Université du Québec à Trois-Rivières, Trois-Rivières, QC, Canada; ^2^ VITAM-Centre de Recherche en Santé Durable, Québec, QC, Canada; ^3^ Centre de Recherche du Centre Intégré de Santé et Services Sociaux de Chaudière-Appalaches, Lévis, QC, Canada; ^4^ Faculty of Medicine, Family and Emergency Medicine Department, Université Laval, Québec, QC, Canada; ^5^ Faculty of Medicine, Centre Intégré Universitaire de Santé et de Services Sociaux de la Mauricie et du Centre-du-Québec Affiliated with Université de Montréal, Trois-Rivières Family Medicine University Clinic, Trois-Rivières, QC, Canada; ^6^ Department of Nursing, Université du Québec à Trois-Rivières, Trois-Rivières, QC, Canada; ^7^ Southwestern Academic Limb Salvage Alliance (SALSA), Department of Surgery, Keck School of Medicine of University of Southern California, Los Angeles, CA, United States

**Keywords:** diabetes, foot ulcer, lower extremity amputation, indigenous peoples, diabetic neuropathy, peripheral arterial disease, health equity

## Abstract

**Introduction:**

Indigenous peoples in Canada face a disproportionate burden of diabetes-related foot complications (DRFC), such as foot ulcers, lower extremity amputations (LEA), and peripheral arterial disease. This scoping review aimed to provide a comprehensive understanding of DRFC among First Nations, Métis, and Inuit peoples in Canada, incorporating an equity lens.

**Methods:**

A scoping review was conducted based on Arksey and O’Malley refined by the Joanna Briggs Institute. The *PROGRESS-Plus* framework was utilized to extract data and incorporate an equity lens. A critical appraisal was performed, and Indigenous stakeholders were consulted for feedback. We identified the incorporation of patient-oriented/centered research (POR).

**Results:**

Of 5,323 records identified, 40 studies were included in the review. The majority of studies focused on First Nations (92%), while representation of the Inuit population was very limited populations (< 3% of studies). LEA was the most studied outcome (76%). Age, gender, ethnicity, and place of residence were the most commonly included variables. Patient-oriented/centered research was mainly included in recent studies (16%). The overall quality of the studies was average. Data synthesis showed a high burden of DRFC among Indigenous populations compared to non-Indigenous populations. Indigenous identity and rural/remote communities were associated with the worse outcomes, particularly major LEA.

**Discussion:**

This study provides a comprehensive understanding of DRFC in Indigenous peoples in Canada of published studies in database. It not only incorporates an equity lens and patient-oriented/centered research but also demonstrates that we need to change our approach. More data is needed to fully understand the burden of DRFC among Indigenous peoples, particularly in the Northern region in Canada where no data are previously available. Western research methods are insufficient to understand the unique situation of Indigenous peoples and it is essential to promote culturally safe and quality healthcare.

**Conclusion:**

Efforts have been made to manage DRFC, but continued attention and support are necessary to address this population’s needs and ensure equitable prevention, access and care that embraces their ways of knowing, being and acting.

**Systematic review registration:**

Open Science Framework https://osf.io/j9pu7, identifier j9pu7.

## 1 Introduction

The estimated population of Canada is 40 million and the diabetes rate is rising (1, 2). Canada’s Constitution Act (1982) recognizes three distinct groups of Indigenous peoples: First Nations, Inuit and Métis, and they account for around 5% in Canada’s total population (3, 4). Approximately 58% of the Indigenous population in Canada identifies as First Nations (5). The demographic of this population is growing rapidly, and young people are more exposed to diabetes and its complications (3, 6–8). Indigenous peoples are affected by type-2 diabetes 3 to 5 times higher than the general population and this chronic disease is one of the fastest increasing health issues among this population (7). Indigenous peoples worldwide, including in Canada, are disproportionately affected by diabetes due to many factors such as genetic predisposition, new environmental exposures, poverty, scarcity of resources and many other barriers that can affect an optimal diabetes care (e.g., geographical isolation, educational status, employment disadvantage, both cultural and linguistic differences) (9, 10). From an Indigenous perspective, rooted in a holistic understanding of health, diabetes is perceived as being associated with the processes of colonization, notably through the loss of traditional ways of life and spirituality, socio-economic marginalization, socio-cultural upheaval, stress and racism (11).

Indeed, Indigenous peoples are diagnosed with diabetes at a younger age, have greater severity of diagnosis, develop higher rates of complications and experience poorer treatment outcomes (12). These outcomes are greater with remote and rural populations (13). Compared to non-First Nations, older First Nations individuals with diabetes are at greater risk of diabetes-specific hospitalization and this can be challenged in regard to ethnocultural considerations and the geographical realities (14). They are also more at-risk of experiencing diabetes-related foot complications (DRFC) such as diabetic foot ulcer (DFU), lower extremity amputations (LEA), infections, foot deformities, Charcot neuroarthropathy, peripheral arterial disease (PAD) and neuropathy (12, 15).

Up to 34% of people with diabetes will develop a DFU during their lifetime which is a significant cause of disabilities, reduces quality of life and can lead to premature death (16). Moreover, LEAs, which are an estimated potential outcome for 1 in 5 DFUs, have an estimated 5-year mortality rate of 51% after a major LEA (16, 17). Personal, societal and economic outcomes of DRFC highlight the importance of supporting prevention strategies for the at-risk population and implementing effective team management approach (18, 19). It is even more important to act towards populations facing at time multiple and intersecting oppression, such as Indigenous peoples, since ethnicity has been identified as a predictor of worse outcomes such as LEAs and health care marginalization (15, 20, 21). It is even more appropriate to talk about the colonization and oppression rather than ethnicity which has led to the worst outcomes for this population and therefore this population has particular cultural needs (22). Thereafter, we refer to indigenous identity and not to ethnicity to respect these peoples. The effect of rurality is also closely associated especially for LEAs (15, 23, 24). Some evidence is published worldwide about diabetic foot disease and DRFC among Indigenous peoples (10, 25, 26), but the specific portrayal of DRFC for Indigenous peoples in Canada is lacking. It is recognized that there are health inequities and disparities as well as poor health care experience for this population (27, 28). Therefore, the aim of this scoping review is to map the existing literature related to diabetic foot disease among Indigenous peoples in Canada based on a western systematic methodology and incorporating an equity lens.

## 2 Methods

The present study will follow the six-stage approach developed by *Arksey and O’Malley* (29), refined by *Levac* and *Colquhoun* (30, 31), and also described by the Joanna Briggs Institute (32). Those stages are mentioned thereafter. Reporting will be compliant with the Preferred Reporting Items for Systematic Reviews and Meta-Analysis Extension for Scoping Reviews (PRISMA-ScR) Checklist (33). The iterative nature of scoping review includes refinement of specific sections of the method as the review progresses. This project has been registered on Open Science Framework (https://osf.io/j9pu7/).

### 2.1 Stage 1: research questions and definitions

#### 2.1.1 Detailed research questions of interest

Based on PICO strategy (34): What are the data regarding diabetes-related foot health outcomes (O) among Indigenous peoples in Canada (P), whether compared to the general population or not (C), for all types of health interventions including epidemiological surveillance data (I). Specific questions were:

- What is the available data on DRFC such as DFU, LEA, diabetic foot infection (DFI) experienced by Indigenous peoples in Canada?
- What is the available data on diabetes foot disease risk factors such as foot deformities, Charcot neuroarthropathy, PAD and neuropathy in Indigenous peoples in Canada?
- What is the available data on diabetic foot disease and DFRC on quality of life and mortality?
- What are other relevant variables such as patient-related outcomes and patient-related experiences related to this topic in Indigenous peoples in Canada?
- Does the reported data on this topic include demographic and equity factors based on the *PROGRESS-Plus* framework (35)?
- Do the included studies report any collaborations and/or partnerships with Indigenous peoples and/or community related to patient-oriented/centered research (36)?

The *PROGRESS-Plus* framework was developed for describing and assessing equity related to the social determinants of health within and across populations (37). Patient-oriented/centered research (POR) can support equity-focused health care research with Indigenous peoples, as the research findings are based on their needs, perspective and context as active stakeholders in the process (36, 38).

#### 2.1.2 Definitions

The broad concept of interest in this study was to identify the burdens of diabetic foot disease/DFRC experienced by Indigenous peoples in Canada. Diabetic foot disease/DFRC were mostly defined by the International Working Group on the Diabetic Foot (IWGDF) criteria and definitions (39). The “diabetic foot ulcer (DFU) “is defined as a break of the skin of the foot, that involves as a minimum the epidermis and part of the dermis, in a person with currently or previously diagnosed with diabetes and usually accompanied by neuropathy and/or peripheral arterial disease within the lower extremity; “neuropathy” is defined as the presence of symptoms or signs of nerve dysfunction in a person (a history of) with diabetes, after the exclusion of other causes. This can also include loss of protective sensation characterized by an inability to sense light pressure (10 g Semmes-Weinstein monofilament); “Peripheral artery disease” (PAD) is defined as an obstructive atherosclerotic vascular disease with clinical symptoms, signs, or abnormalities on non-invasive or invasive vascular assessment, resulting in disturbed or impaired circulation in one or more extremities. This can cause claudication and rest pain. “Infection” is defined as a pathological state caused by invasion and multiplication of microorganisms in host tissues accompanied by tissue destruction and/or a host inflammatory response; “lower extremity amputations (LEA)” is defined as a resection of a segment of a limb through a bone or through a joint; Charcot neuroarthropathy (Charcot foot) is a non-infectious destruction of bone(s) and joint(s) associated with neuropathy, which, in the acute phase, is associated with signs of inflammation (39). “Foot deformities” are defined as structural and functional foot deformities occurring with diabetes and motor neuropathy causing atrophy and muscle imbalances such as claw and hammer toes, prominent metatarsal heads, *pes cavus*, *pes equinus*, hallux *limitus* or *rigidus* and *hallux abductovalgus* (40). The Western “health-related quality of life” refers to an individual’s perception of their position in life in the context of the culture and value systems in which they live and in relation to their goals, expectations, standards and concerns. It is a broad-ranging concept affected in a complex way by the individual’s physical health, psychological state, level of independence, social relationships, and their relationships to salient features of their environment as they relate to the DRFC context (41).

### 2.2 Stage 2: identifying relevant studies

The search protocol and strategies were developed by two members of the research team (VB and JP) and revised by another team member (MBF). The primary information source included systematic search from the following database: 1) MEDLINE, 2) CINAHL, 3) EMBASE, 4) Cochrane Library, 5) Native Health Database, 6) Government Health Indigenous Affairs Departments of the United States/Canada and 7) LiSSa. The secondary information source included reference lists as well as citation searches of related relevant citations. Canadian clinical guidelines from major organizations with an interest towards in diabetic population were reviewed. Grey literature was assessed through Google Scholar, Open Access Theses and Dissertations, ProQuest, ClinicalTrials.gov and Réseau Santécom. The search strategy, limited to articles in English and French, was developed for MEDLINE database (**Supplement Material 1**), with the assistance of a qualified librarian and involved a combination of key terms and concepts (MeSH, non-MeSH, key terms and free vocabulary). The search strategy was adapted for other databases and identical terms translated to French were used to search in selected French-language databases. This review had searched articles from inception up to August 29^th^, 2022. Citations from all information sources were merged and duplicates removed using EndNote (version 20.4, Clarivate Analytics, 2022).

### 2.3 Stage 3: study selection and criteria

Two independent reviewers (VB and JP) initially met to clarify the following inclusion criteria:

- Population: Adult (18 years and older) Indigenous peoples in Canada with either type-1 or type-2 diabetes with any DRFC or disease;
- Intervention: Any interventions including none;
- Comparator(s)/control: Other populations or none;
- Outcomes: Results pertaining or describing data about DRFC on DFU, LEA, DFI, quality of life, mortality, foot deformities, Charcot foot, PAD, neuropathy or other relevant data about DRFC (e.g., DFU recurrence, genetics, etc.);
- Settings: Any clinical settings or community;
- Languages: English or French

Exclusion criteria were:

- Publication/study design: Conference or meeting abstracts, commentaries, letters and correspondences, Editor’s response, protocol descriptions;
- Population: Individuals who were not considered as Indigenous in Canada (e.g., native from other countries); gestational diabetes; wounds, amputation or death in the absence of a diagnosis of either type-1 or type-2 diabetes.

The search strategy was completed by one of the authors (VB). Two arms of reviewers (VB/JP and VB/SL) have independently screened titles and abstracts using eligibility criteria. Then, relevant papers were read entirely, and eligibility criteria were systematically applied. Disagreement was settled using a consensus approach between reviewers and a third person intervened if required (MBF). Eligibility criteria were clarified following a training exercise on the first 300 citations and inter-rater agreement (kappa statistic) was greater than k=0.70, signifying substantial agreement, and then selection was completed (42).

### 2.4 Stage 4: charting the data

A Microsoft Excel (Microsoft Corporation) spreadsheet served as the data extraction form developed by the two reviewers (VB and AML) and updated by an iterative manner during the full article revision process. The data-charting form includes the PICO elements, “*PROGRESS-PLUS*” factors (place of residence, race/ethnicity/identity/culture/language, occupation, gender/sex, religion, education, socioeconomic status, and social capital) including age and disabilities for the equity lens, year of publication, authors, study location, study design, type of data, sample sizes, aims of study and important results extracted from selected articles (35). We also identified whether a patient-oriented research strategy was integrated or not. The extraction of all information was conducted by one reviewer (VB) and double-checked by one of two reviewers (JP and SL).

### 2.5 Stage 5: collating, summarizing and reporting results

A visual flow diagram (PRISMA) outlined the decision-making in the study selection process (33). Frequency measures such as numbers and their percentages numerical summary for the overall study characteristics and a narrative synthesis was conducted, centered on every variable aimed to answer our research sub-questions on Indigenous peoples in Canada. We have also aggregated the results in tables to identify the elements associated with equity, the integration of patient-oriented research, and the key findings from included studies. Risk of bias assessment is not mandatory in a scoping review, as many different study designs are included. However, two reviewers (VB and ST) conducted an appraisal based mixed methods appraisal tool (MMAT) and chose *at posteriori* according to the studies included (43).

### 2.6 Stage 6: consultation

Even though consultation of knowledge users (e.g., clinicians, citizens, patients and caregivers, decision makers, other researchers) is optional, it enhances the methodological rigor and the validity of the review. Thus, to gain appreciation of the review’s findings, the lead reviewer (VB) approached diabetic foot disease and Indigenous stakeholders in Canada to provide voluntary insights about our review.

## 3 Results

### 3.1 Search and selection

**Figure 1:**
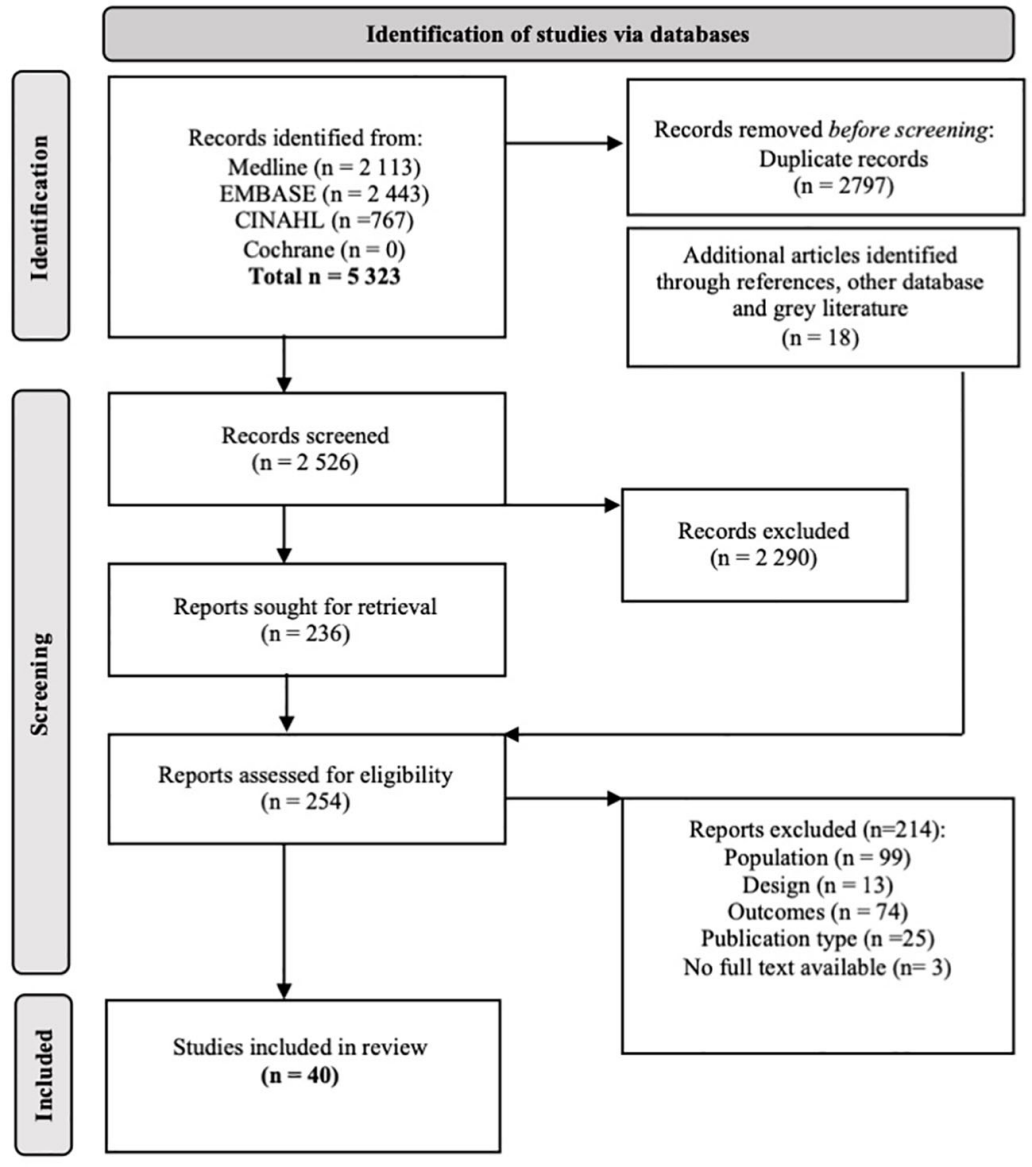
Study selection flowchart. Adapted from Page MJ, McKenzie JE, Bossuyt PM, et al. The PRISMA 2020 statement: an updated guideline for reporting systematic reviews. BMJ. 2021;372:n71. Open Access.

A total of 5, 323 records were identified from bibliographic databases and 18 from additional searches. All duplicates were removed with Endnote and 2, 526 titles were screened. We retained 40 studies that reported data on diabetic foot disease for Indigenous peoples in Canada, as represented in the flow diagram (**Figure 1**) (44–81). Some included studies have described similar dataset/population [(75, 79) (80, 81) (48, 64) (44, 82); and (49, 78)] and therefore, they were merged and reported together for a same study design/similar outcomes or separately for significant differences (13, 52) and (50, 60) (**Table 1**).

**Table 1:** Overview of included studies (n= 40).

First author	Year	Study Design	Data	Canadian Location	Indigenous people			DFU	LEA	N	PAD	FD/C	M	DFI	QoL	ORV
					FN	M	I									
Chan	2021	CS	Administrative Database	On	√				√							
Essien^†^	2020; 2021	CS	Administrative Database	SK	√				√		√					
Pace^†^	2020	CS	Registry Data	BC, AB, MB, ON, QC, NF	√					√						
Hayward^†^	2020	MMS	Workshops, Registry data, Chart Review	BC, AB, MB, ON, QC, NF	√					√						
Shah	2019	CSS	Administrative Database	ON	√				√		√		√			√
Loewen	2017	CS	Census and Administrative Database	ON	√				√							
Turin	2016	CS	Administrative Database	AB	√						√					
Al Sayah	2015	CSS from CS	Self-Reported (self-administered questionnaires); Administrative Database	AB	NA	NA	NA	√	√	√	√			√		
Maple-Brown^†^	2012	CSS	Data from Hanley 2005	ON	√			√	√	√	√					
Martens^†^	2010 updated in 2012; 2002	CS	Administrative Database	MB		√			√							
Reda	2012	CSS	Chart Review	MB	√	√		√	√	√	√	√				
Harris	2011	CSS	Chart Review	BC, AB, SK, MB, ON, QC, NF	√				√	√	√					√
Oster	2010	CS	Physical Examination	AB	√											√
Shah^†^	2010; 2011	CSS	Administrative Database	ON		√			√							√
Lovell	2009	CSS	Self-Reported (Phone survey)	NA - pancanadian	√						√					
Oster	2009	CSS	Self-Reported	AB	√			√		√						√
Bruce	2008	CSS	Physical Examination	MB	√			√	√	√						
Dannenbaum	2008	CSS	Administrative Data	QC	√			√	√	√	√					
Attawar	2006	Q	Interviews, Registry Data, Physical Examination	MB	√			√	√	√	√	√			√	
Virani	2006	CS	Questionnaire, Physical Examination, Chart Review	AB	√											√
Martens^†^	2007	CSS	Administrative Database	MB	√				√							
McIntyre	2007	CSS	Chart Review, Physical Examination and Interviews	MB	√	√		√	√	√	√	√	√	√		√
Rose	2007	CSS	Chart Review	MB	√	√		√	√							√
Goulet	2006	CSS	Chart Review	MB	√	√	√		√		√					√
Reid	2006	CSS	Interview, Physical Examination, Chart Review	MB	√	√		√	√	√	√	√				√
Hanley	2005	CSS	Physical Examination, Laboratory Analysis, Questionnaire	ON	√					√	√					
Meatherall	2005	MMS	Chart Review and Questionnaire	MB	√	√			√						√	
Pollex	2005	CSS	Physical Examination, Self-Reported, and Laboratory	ON	√						√					
Iwasaki	2004	Q	Focus Groups	MB	√	√										
Légaré	2004	CSS	Administrative Database, Registery	QC	√				√							
Thommasen	2004	CSS	Chart Review	BC	√					√	√					
Jin	2002	CSS	Administrative Database	BC	√					√						
Hernandez	1999	Q	Interview	ON	√				√				√			
Brassard	1995	CSS	Chart Review	QC	√					√	√					√
Macaulay	1988	CSS	Chart Review, Interview and Physical Examination	QC	√				√	√	√					
Young	1985	CSS	Administrative Database and Charts Review	MB, ON	√					√						

^†^Similar population/dataset (Essien 2020 and 2021; Pace 2020 and Hayward, 2020; Mapple-Browns 2012 and Hanley 2005 ; Martens 2002, 2007, 2010 and 2012; Shah 2010 and 2011)

MMS, Mixed-method study; CSS, Cross-sectional study; Q, Qualitative study; CS, Cohort study; MB, Manitoba; QC, Québec; SK, Saskatchewan; ON, Ontario; BC, British Colombia; AB, Alberta; NF, Newfoundland and Labrador; FN, First Nation; M, Métis; I, Inuit; NA, Not available; DFU, Diabetic foot ulcer; LEA, Lower extremity amputation; N, Neuropathy; PAD, Peripheral arterial disease; FD/C, Foot deformities or Charcot; M, Mortality; DFI, Diabetic foot infection; QoL, Quality of life; ORV, Other relevant variables.

√ means that it fits the category of the colon.

### 3.2 General characteristics of included studies and population

General characteristics of the included studies and population are presented in the **Table 1**. The studies were published between 1985 and 2021. Most (51%) were published between 2000 and 2010 (52–70, 76, 82) and between 2010 and 2021 (39%) (44–51, 75, 77–79). Five percent were published between 1985 and 1990 (73, 74) and another five percent between 1991 and 2000 (71, 72). The majority of the studies were quantitative (86%), with the majority (61%) using a descriptive cross-sectional design (44, 47, 48, 50, 51, 53–57, 59–64, 66, 68–70, 72–74, 76) and the remainder (22%) using an observational cohort design (45, 46, 49, 52, 75–77, 80). There were also three qualitative studies (8%) (58, 67, 71) and two mixed-method studies (6%) (65, 81). Various types of data were reported such as administrative data (44, 46, 49, 53, 57, 59, 68, 70, 74, 75, 77, 79) and registry (58, 80, 81), self-reported data (47, 54, 55, 66), retrospective chart review data (50, 51, 61–63, 65, 69, 72–74), prospective data (e.g., physical examination, interviews, focus, questionnaires, etc.) (52, 56, 58, 60, 63–67, 71, 73, 76, 81) and data from a previous prospective study (48). The majority of studies were conducted in Indigenous population in Manitoba, followed by those living in Ontario. One study was pan-Canadian and the provinces and territories were not specified (54). No study included the Yukon, Nunavut and the Northwest Territories population. The Atlantic region was poorly represented with only three studies (but two with the same dataset) that included Newfoundland and Labrador in the overall study (51, 80, 81). The situation was similar for Saskatchewan (51, 75, 79).

Of the 40 studies included, the most published data were focused on First Nations (92%). Only one study (3%) did not distinguish specifically the identity of its population (i.e., First Nations, Métis, or Inuit) (47). Inuit were less represented, being included in only in one study (3%) (62). Métis were represented in a quarter of the studies (26%) (49, 50, 53, 60–63, 65, 67). All studies included at least 332,233 individuals from Indigenous peoples in Canada. The residential area of the community was mentioned for only 53% (19/36) of included studies from which eight studies clearly mentioned the population living on communities (i.e., on reserve) (52, 55, 56, 59, 64, 68, 77, 80, 81). Ten studies did not report demographic data about population with diabetes and/or Indigenous peoples only (47, 49, 50, 54, 62, 63, 66, 69, 70, 79). All details about the population are presented in **Supplemental Material 2**.

### 3.3 Diabetes foot disease and complications outcomes

Nine studies did not present clear outcomes: microvascular disease including neuropathy, retinopathy and nephropathy (80, 81), surgery for leg circulation, including LEA (77), other atherosclerosis (including gangrene and other peripheral vascular disease) and neuropathy and amyotrophy (70), PAD (including ischemic feet, LEA and claudication) (73), microvascular disease including ischemic heart disease, cerebrovascular disease and peripheral vascular disease (72), ulcers or sores on their feet and legs (55), foot or leg ulcers or infection/gangrene or LEA (47) and precise type of amputation (i.e., lower, upper, traumatic, etc.) not mentioned (51). Therefore, their data are detailed in the **Supplemental Material 2**. and sparsely integrated. Studies that reported results for Indigenous peoples with comparators are presented in **Table 2**. The main results are also summarized in the following subsections.

**Table 2:** Major findings concerning diabetic foot outcomes for Indigenous peoples^†^.

Author, Year	Trends for the Indigenous peoples	Comparator
Essien, 2020;2021^‡^	↑: Overall LEA rate*; Primary LEA*; Subsequent LEA*; Major LEA*; Minor LEA* ↑: Post-operative acute care length of stay* Age-adjusted ↑: LEA rate for people aged of 50 years and over for both population; LEA rate* Sex-Adjusted ↑: LEA rate for males in both population Indigenous female almost twice likely to have a LEA* Indigenous male at higher risk*	Non-Indigenous Population with or without diabetes; Population with LEA
Shah, 2019	Number of revascularization procedures are comparable, but PAD may be underdiagnosed. ↑: LEAs are 3-5 times higher; For people aged of ≤ 44 years: LEA are 6 times more frequent; LEA rates associated with increased age and rurality. Remote community is associated with LEA in both populations. ↑: LEAs in Indigenous female* than non-indigenous female*; LEAs in Male* ↑: 15% of mortality* Disparity associated with poor access to care, specialized services for wounds care and rehabilitation	Non-Indigenous population
Loewen, 2017	LEA rate is 7 times the provincial rate* Rate for major LEA (below-knee amputation) is 3 times higher* at a lower age	Non-Indigenous population with diabetes data available
Turin, 2016	↓: PAD (0.2% vs. 0.6%)	Non-Indigenous population
Maple-Brown, 2012	↑: Neuropathy*	Other Indigenous population (Australia)
Martens, 2010;2012^‡,^2002	↑: LEA rate*, but similar risk of LEAs with controlled age, sex, income, geographic area, mental and physical comorbidities, continuity of care ↑ similar LEA risk for males, older, living in neighborhood income areas and for those with comorbidities for both population ↓: LEA rate when seeing the same physician for a 2-year periods for both population* ↓: LEA risk associated with continuity of care* 2002: twice ↑ LEA rate for Metis* compared to other Indigenous population and 30 times the non-Indigenous population	Non-Indigenous population with diabetes; Other Indigenous population
Shah, 2010	LEA rate (adjusted sex and age) comparable	Non-Indigenous population with diabetes
Martens, 2007	↑: LEA adjusted sex/age rate* and even more ↑ in some community (Dakota Ojibway Tribal Council) * LEA rate is inversely proportional to specialist access	Non-Indigenous population with diabetes; between the indigenous communities of Manitoba
McIntyre, 2007	Absent Dorsalis Pedis pulse* ↑: History of DFU*, Charcot Foot*, mean number of DFU*, DFU with prior osteomyelitis* ↑: Number of LEA* Reason for inadequate foot care: financial cost, lack of family support, language barrier Risks associated with mortality for both population: number of DFU in the patient history, the proportion of patients with either: an absent dorsalis pedis pulse, prior myocardial infarction, LEA, prior angiogram, not performing a daily foot inspection, occluded vessel detected by angiography	Non-Indigenous population (Caucasians, Filipinos, Asians, east Indians, Blacks)
Rose, 2007	↓: Time from initial visit to major LEA*; also correlated with living in rural or remote communities* Survival time without LEA: risk factors associated with indigenous ethnicity, non-urban residence, and PAD Indigenous ethnicity is not associated with risk factors for poor DFU outcomes Indigenous patients with a DFU had a LEA approximately 12 weeks earlier than in non-indigenous patient with a DFU	Non-Indigenous population (Caucasians)
Goulet, 2006^‡^	↑ Indigenous people with PAD and diabetes required revascularization bybass*, risk factors are lower age* and end-stage renal disease* Indications for bypass procedure: rest pain*, claudication*, gangrene*, non-healing DFU*, acute ischemia ↑ LEA (at the revascularization procedure): at least one toe*, forefoot* Complications after revascularization are not significant (limb loss, wound infection, death)	Non-Indigenous population
Pollex, 2005^‡^	No significant association between MTHFR genotype and intermittent claudication ↑ PAD: Gene MTHFR 677T carriers (677T allele*)	Other Indigenous population (without the gene)
Jin, 2002^‡^	Trends to ↑ neuropathy, amyotrophy, hospitalization, atherosclerosis (including gangrene and PAD)	Non-Indigenous population
Macauley, 1988	↑ PAD (including ischemic foot, amputation, claudication)* Low rate of neuropathy	Indigenous population without diabetes (Mohawks)

^†^Studies without comparative groups/data were excluded (n=10) ; Studies with not clear outcomes were excluded (n= 4).

^‡^Not specific data for the population with diabetes type 2 only and/or indigenous people only (n = 10).

*Statistically significant.

↑, Augmentation/Increase.

↓, Diminution/Decrease.

LEA, Lower Extremity amputation; PAD, Peripheral Arterial Disease; DFU, Diabetes Foot Ulcer.

#### 3.3.1 Diabetic foot ulcer

Ten studies provided data on DFUs (**Table 1**) (47, 48, 50, 55–58, 60, 61, 63). The prevalence of DFU ranged from 1% to 39% (47, 48, 50, 55, 57, 58, 60), and the prevalence of a history of DFU from 32% to 75% (58, 60). Only one person had a history of DFU in a population of 483 First Nations people (56). Six to fifteen percent of individuals had a history of DFU or had active DFU (13, 63).

#### 3.3.2 Lower extremity amputation

Twenty-four studies provided data about LEAs, and thus it is the most studied outcome (44, 46–51, 53, 56–63, 65, 68, 71, 73, 75, 77–79). The incidence of LEA varied among communities and was estimated to range between 1.19 à 6.16 per 1,000 persons, and rates of LEA were inversely related to the access to specialists (59). The prevalence was estimated between 0 and 36% in this population (48, 49, 51, 57, 60). Studies have estimated that the prevalence of LEA was 7 to 49 times higher than the Indigenous population with diabetes than in the non-Indigenous population without diabetes (46, 49, 58, 78). A study identified that LEA’s frequency was 3 to 5 times higher to the non-Indigenous comparative across sex, age and location (44). Among people with diabetes, Ethnicity, or colonization, as experienced by people identifying as Indigenous would lead to a 1.7-fold increase in the risk of having LEA (75). Specifically, in the Métis population, the sex- and age-standardized LEA rate was equivalent to that of the entire population with diabetes (82). Their risk of LEA was similar compared to other Manitobans after controlling sex, age, income, geographic area, mental and physical comorbidities and continuity of care (49). However, a higher risk of LEA was identified in Métis male *vs*. female (59).

The risk factors for LEAs, similarly to non-Indigenous peoples, were male sex, living in low-income area, living with comorbidities, and being older. A protective factor was to see the same physician for at least one half of their visit over the two-year period (49, 75). Among those aged 44 years or younger, the frequency of LEA was six times higher and living in a remote community was a high-risk factor for LEA (44). The first major LEA on Indigenous peoples occurred at a younger age (65), Indigenous peoples had a shorter average time from initial clinic visits to major LEA compared to non-Indigenous population which also correlated with living on rural or on reserve (61). When controlling the effect of the place of residence (i.e., rurality and on reserve), Indigenous identity was not associated with poorer outcomes such as LEA and death, but early LEA was associated with non-urban residence, identity and arterial insufficiency (61). Indigenous patients with DFU are at-risk of LEA approximately 12 weeks earlier than non-Indigenous patients (61). A study has reported that on average, Indigenous peoples had less phantom limb pain (65). A study related to diabetes has observed seven hospitalizations, totalizing 81 days of hospitalization over a 5-year period related to five cases of amputations (68). Finally, LEA trends (i.e., overall LEA rate, primary LEA, subsequent LEA) increased over a 13-year period by about 5% over this period compared to the trend in the non-Indigenous population which was more stable or declining (75).

#### 3.3.3 Neuropathy

Eighteen studies provided data about neuropathy (13, 47, 48, 50, 51, 56, 58, 63, 64, 69, 70, 72–74, 80, 81). The prevalence of neuropathy ranged from 5% to 94% (47, 56, 58, 60, 63, 64, 72–74). Prevalence of neuropathy was reported higher in Indigenous peoples in Canada compared to the one from Australia (48). Among Indigenous peoples, the likelihood of developing neuropathy was 2.7 times higher for women than for men and 3 times higher for those who had completed less education than for those who had completed grade 9 or higher. The risk of neuropathy was twice as high for a person with a glycated hemoglobin level of 9% compared to 6%, and 3 times higher for heavy smokers (56).

#### 3.3.4 Peripheral arterial disease

Eighteen studies provided data about PAD (44, 45, 47, 48, 50, 51, 54, 57, 58, 60, 62–64, 66, 69, 72, 73, 75). PAD prevalence was estimated between 0.2% to 23.0% (47, 48, 51, 57, 64, 69, 75). A genetic mutation that may be present particularly in Indigenous peoples is significantly associated with an increased risk of PAD (66). It was found that 92% of Indigenous peoples with diabetes and PAD required more bypass revascularization compared with 42% in the non-Indigenous population (62). Indigenous peoples also had a greater burden of PAD symptoms (i.e., claudication, rest pain, gangrene and acute ischemia) than the non-Indigenous (62).

#### 3.3.5 Foot deformities and charcot neuroarthropathy

Four studies provided data related to foot deformities and Charcot neuroarthropathy (50, 58, 60, 63). Foot deformities were estimated to be between 16% and 51% in Indigenous peoples and included hallux valgus, claw toes, hallux rigidus, flat feet, cavus feet, long second toe, ankle deformity, heel pad atrophy, dorsal exostosis (58, 63). There was more Charcot neuroarthropathy in the Indigenous group with end-stage renal disease than in a similar non-Indigenous group (60). In a study, Charcot neuroarthropathy was a very rare condition estimated at less than 1% of the population (63).

#### 3.3.6 Mortality

Three studies provided data related to mortality and DRFC (44, 60, 71). Mortality (age- and sex- adjusted) after LEAs was 15% higher in Indigenous peoples than in non-Indigenous peoples, with a median survival of 3.5 years compared to 4.1 (44). First Nations people with diabetes were very concerned about the loss of freedom, mortality and LEA (71). Risk factors for mortality were the same for the Indigenous and comparative populations i.e. mean number of prior DFU, the proportion of patients with either an absent dorsalis pedis pulse, prior myocardial infarction, LEA, prior angiogram, not performing a daily foot inspection, occluded vessels detected by angiography (60).

#### 3.3.7 Diabetic foot infection

Two studies provided data related diabetic foot infections (47, 60). In patients with diabetes and end-stage renal disease, DFUs had significantly greater prior osteomyelitis amongst Indigenous peoples compared to their non-Indigenous counterparts (60).

#### 3.3.8 Quality of life

Three studies provided data related to diabetic foot disease and quality of life (58, 65, 67). Indigenous peoples reported having suffered from LEA for the rest of their life and living in fear of the future (for themselves and their families). They realized that they have to live with these DRFC on a daily basis and were stressed about living another 20 years because they realized that it may get worse (67). There was no difference between Indigenous and non-Indigenous peoples in their feelings of distress related to DRFC. They expressed feelings of regret, self-blame, and guilt about their general health, diabetes, and LEA (65). Many Indigenous peoples reported chronic and persistent foot pain, which affected their quality of life. LEA has changed their lives as it restricted their ability to participate meaningfully in their community (58).

#### 3.3.9 Other relevant data

There were 11 studies that provided insights about other relevant data detailed in **Supplemental Material 2**. Over a 7-year period, for 169 people with 498 DRFC, this resulted in 18% emergency room visits, 16% hospitalizations, 11% elective transfers and 6% emergency transfers (63). Progression of poor clinical outcomes in this population is associated with referral with a lesion, age greater than 60 years, prior LEA or vascularization, PAD, more than one lesion at presentation, longer duration of diabetes, higher grade of DFU on the Wagner classification (61). The reasons for inadequate foot care are associated with financial cost, lack of family support, and language barriers (60). Despite interventions to achieve the recommended practice guidelines and recommendations, there is still limited foot screening in this population (76, 81). Foot abnormalities are more common in Indigenous men (52), and unspecified diabetic foot disease was estimated to 35% of the population from 19 different Indigenous communities (51). The revascularization rate (age and sex adjusted) is equivalent to the non-Indigenous and Indigenous populations with diabetes (82), and the Indigenous population presented worsen symptoms before revascularization (62). Health disparity related to DRFC in Indigenous population may be driven in part by poor access to health care, particularly specialized services for wound care and rehabilitation and especially because of their residency in remote communities (82). Indigenous identity is associated with prolonged postoperative acute care length of stay after a LEA (79).

### 3.4 Equity lens and patient-oriented research

We have listed the different factors of the *PROGRESS-Plus* framework in **Table 3** for the included studies. The most frequently included factors in ascending order were place of residence, sex, race and age. One study included all factors (58). Education, income, and social per capita were minimally included in the included studies. There was little data on the occupation of the Indigenous population. Religion and spirituality were not discussed in any study. Patient-oriented research has been clearly mentioned in six studies representing 17% of included studies (49, 51, 58, 75, 77–79, 81), and it was particularly favored in the last decade. Details about the integration of patient-oriented research are also displayed in **Table 3**. None of those studies has used the *GRIPP-2* tool to reported patient and public involvement in research (83).

**Table 3:** PROGRESS-Plus Factors and Patient-Oriented Research Data in the Included Studies.

Author,Year	P	R	O	G	R	E	S1	S2	+	Details *plus* factors	POR	Comments regarding POR
Chan, 2021										Age		Data planification and collection; Consent of the leader of the community to publish data.
Essien, 2020;2021										Age		Multidisciplinary patient-oriented research team comprised of people with amputation, caregivers, researchers, educators, and health care providers. Not stated if people from Indigenous communities.
Pace, 2020										Age		Not reported
Hayward, 2020										Age		Integrated community as partners in developing culturally relevant innovations and improved care/access. FORGE-AHEAD is co-designed.
Shah, 2019										Age		Not reported
Loewen, 2017										Age		Not reported
Turin, 2016										Age		Not reported
Al Sayah, 2015										Age		Not reported
Maple-Brown, 2012										Age		Not reported
Martens, 2010;2012; 2002										Age		Wellness-lens approach and partnership; Culturally coherent; Holistic approach to knowledge translation
Reda, 2012										Disability: hemodialysis/ end-stage renal disease		Not reported
Harris, 2011										Age		Community participation after community consultations (data collection)
Oster, 2010										Age		Uncertain. SLICK program is in collaboration with First nations.
Shah, 2010												Not reported
Lovell, 2009												Not reported
Oster, 2009										Age		Uncertain. SLICK program is in collaboration with First nations.
Bruce, 2008										Age		Not reported
Dannenbaum, 2008										Age		Not reported
Attawar, 2006										Age		Integrating fundamental principles of community-based participatory research, collaboration, equity, community development, and action); conducted in collaboration with First Nation communities in Manitoba and supported by community diabetes research working group.
Virani, 2006										Age		Uncertain. SLICK program is in collaboration with First nations.
Martens, 2007										Age		Not reported
McIntyre, 2007										Age; Disability: hemodialysis/end-stage renal disease		Not reported
Rose, 2007										Age		Not reported
Goulet, 2006										Age; Disability: need revascularization		Not reported
Reid, 2006										Age		Not reported
Hanley, 2005										Age		Not reported
Meatherall, 2005										Age; not receiving dialysis		Not reported
Pollex, 2005										Age; Disability: genetic predisposition PAD		Not reported
Iwasaki, 2004										Age		Not reported
Légaré, 2004										Age		Not reported
Thommasen, 2004												Not reported
Jin, 2002										Age		Not reported
Hernandez, 1999										Age		Not reported
Brassard, 1995										Age		Not reported
Macauley, 1988										Age		Not reported
Young, 1985										Age		Not reported

The shaded boxes indicate the presence of this factor.

P, place of residence; R, race/ethnicity/culture/language; O, occupation; G, gender/sex; R, religion; E, education; S1, socioeconomic status; S2, social capital; + , Plus Factors; POR, Patient-oriented Research

### 3.5 Bias

**Figure 2:**
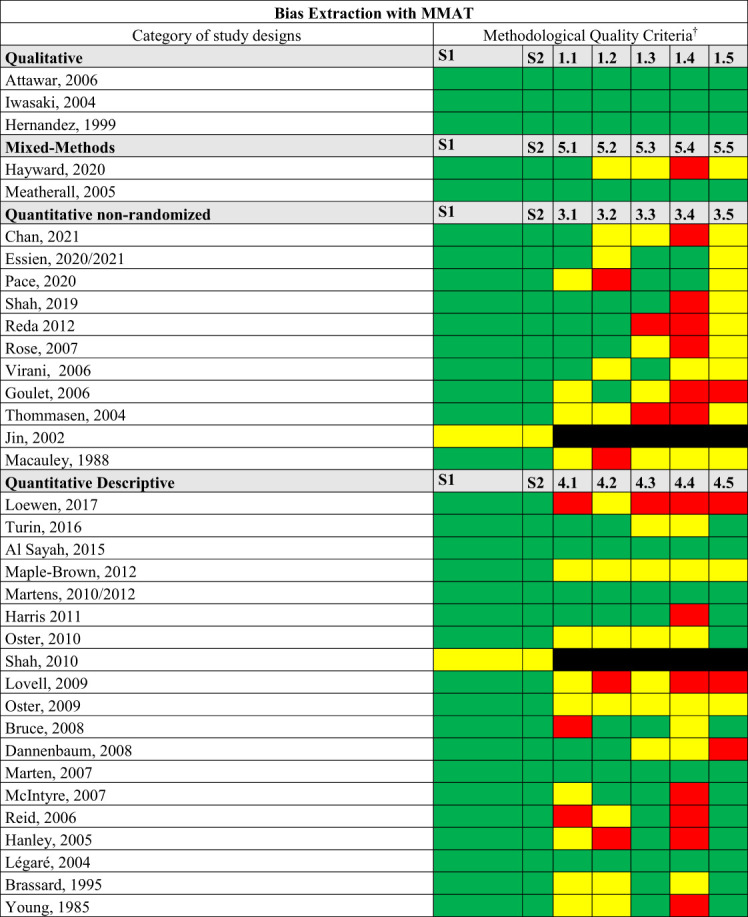
Quality Appraisal of included studies. MMAT, Mixed Methods Appraisal Tool. †Are criteria are available in MMAT (84).

We conducted a critical quality appraisal of the included studies, and the results are presented in **Figure 2**. All three qualitative studies were of good quality. One of the mixed-method studies was good (81). The quality of the non-randomized qualitative and descriptive studies was variable but mostly of average quality. It was highlighted by the consultation that there is substantial bias in that the bulk, if not all, studies included were led by non-Indigenous people using non-Indigenous methodology.

### 3.6 Consultation

Six stakeholders from the Indigenous peoples and/or working very closely with them were consulted about this review: a citizen, a patient with DRFC, a caregiver, a clinician, a decision-maker and a researcher. These people chosen from our networks are from different communities and representing three different provinces. Their feedback was incorporated into this review.

## 4 Discussion

Our objective was to map the existing literature published in database related to diabetic foot disease among Indigenous peoples in Canada based on a systematic methodology and incorporating an equity lens. Thus, Indigenous peoples experience a heavy burden of diabetic foot disease compared to the non-Indigenous population. LEA, the most reported complications, are higher in Indigenous peoples. Very little is reported on patient-reported experience and outcomes related to DRFC. Besides, studies mainly report on First Nations and Métis data, with very little representation for Inuit people. Data on Inuit living with diabetes in Northern communities in Canada is limited, as they represent the least populated group in the Indigenous population (3). Their voices are still less represented in diabetes research which is coherent with our results (85). We examined our results from three perspectives: trends in DRFC, the equity lens, and POR.

### 4.1 Trends in diabetes-related foot complications and diseases

The trends identified in this study confirm a high level of DRFC in this population, but it may be only the tips of the iceberg. DRFCs affect more Indigenous men than Indigenous women and both sexes are at higher risk for LEA at a younger age than non-Indigenous people. In addition, there is a significant effect of the place of residence where rural and remote communities are associated with increased numbers of LEA. These trends are consistent with those demonstrated previously (10, 86, 87). Indigenous identity is associated with LEA in Indigenous peoples of Australia (88). Recent studies in the United States on race and rurality have identified their association resulting in more LEA events, both major and minor (24, 89). Indeed, deficiencies of specialized care and the effect of rurality on LEA was also demonstrated similarly as highlighted in our review (24). LEAs are amplified by race, particularly in ethnic minorities groups of a population (24). In general, LEAs are also more prevalent among men (90), and this trend was also identified amongst the Indigenous population in Canada. Similarly, the same trend was identified with respect to age, with the mean age of first LEA being younger among Indigenous peoples, approximately 14 years younger than in the non-Indigenous population, and LEA being more common among those under 50 years of age (10, 86). PAD appears to be underdiagnosed in Indigenous peoples, whereas revascularization procedures may be overdone compared with non-Indigenous peoples. Those trends have also been identified among marginalized groups (87, 91). There is very little Canadian data on diabetic foot infection which has been documented to be very prevalent in the Indigenous peoples of Australia (86). Indigenous identity has been associated with an increased risk of neuropathy and DFU, with a 3- to 6-fold increase in the likelihood of experiencing LEA, but our data do not permit such a precise estimate in comparison (86).

Our results support that, although this research topic is receiving more recent attention in Canada, knowledge remains limited. In fact, most of the DFRC identified seem to be underestimated including neuropathy, PAD, diabetic foot infection, when compared to those of the Indigenous population in Australia (86). Moreover, we did not identify any study reporting on mental health (e.g., depression, anxiety) and DRFC. Yet the association with DFU and LEA is well demonstrated (92, 93). This result is consistent with the fact that these data are often missing for this population related to mental health studies (94). Nevertheless, this is a difficult topic for this population given the intergenerational effects of colonization, residential schools and other trauma (95), in addition to competing health priorities.

The overall results are consistent with those of a previous study conducted 10 years ago, which identified increased biomedical risk factors for all Indigenous populations with diabetes related to LEA, neuropathy and PAD, and highlighted that complex political and social factors are also barriers to optimal health care for Indigenous peoples (10). Therefore, Indigenous identity alone does not explain all the outcomes, it is mainly the synergy of socio-historical-political conditions (and colonization) faced by Indigenous peoples that predispose them to diabetes and its complications in Canada (8). Hence the importance of considering factors related to the inequity.

### 4.2 Equity lens and care

This review suggests that the magnitude of the problems associated with diabetic foot disease and its complications in this population is identified but underestimated, particularly with respect to equity as their influence on DRFC remains unclear. That this review did not identify the real inequity experienced by Indigenous people regarding DRFC only highlights how problematic western methodologies are. Only minimal robust data was available, and few studies have incorporated *PROGRESS-Plus* factor perspective. When equity factors are less accounted for in research, this inevitably impacts the results. Yet the effect of equity factors is well known in the Indigenous population with diabetes (12). Strategies were suggested to address social barriers and to improve outcomes, equity and cultural safety approach in Indigenous population in Canada (96). However, it takes time to set up at all levels i.e., individual, organizational, system and in research.

Social determinants of health, identified with *PROGRESS-Plus* factors may not be enough and appropriate. This must be grounded in decolonization and increasingly centering on Indigenous ways of knowing, being and doing and Indigenous health determinants (97). While our project has shown that even Western factors have been given little consideration, it is essential to also include determinants of wellness that are much more aligned with the beliefs, values and preferences of Indigenous peoples, including elements such as self-determination, identity, language and land (98). Mental, physical, spiritual and social are holistic dimensions of health for this population and specific Indigenous frameworks may better support the equity lens (99). Foot health in Indigenous peoples should be no different from the non-Indigenous population and based on prevention and management that is proven to be effective (100). However, evidence-based, trauma-informed, and culturally safe care should be inseparable in order to decrease health disparities for this population. Poor outcomes included in this review may be consistent with the limitations of the Canadian health services/system, especially when actions are not relevant to the social and cultural contexts of Indigenous peoples (12). A focus on building relationships with an Indigenous person with diabetes is important rather than a singular emphasis on achieving management targets. This also needs to be considered in research. Previous studies have shown that there is little good quality evidence to assess diabetes health outcomes in primary care or system services for Indigenous peoples in Canada with type 2 diabetes (101). In addition, the limited success in achieving evidence-based targets (e.g., glycated hemoglobin, lipid levels, physical activity levels) in this population has highlighted the limitations of health services, as the targets are not necessarily relevant to Indigenous peoples and are not aligned with the equity factor (102, 103). The access to culturally safe health care, delivered by culturally competent (allied) health professionals were seen as a contributing factor to foot and lower extremity health (102). This is also aligned with a call to action as per the Truth and Reconciliation Committee of 2015, commissioned by the Government of Canada (104).

Our results highlighted the hypothesis of disparities regarding prevention, treatments, and quality of care, particularly in rural and remote communities, and may be the direct effect of colonization. Disparities have been well demonstrated for the management of diabetes and its complications in rurality (105). Deficiencies of specialized care and the effect of rurality on LEA was demonstrated on race and ethnicity in a previous study (24). Socio-economic conditions and risk factors for type 2 diabetes and its complication are important determinants of health and therefore culturally safe and appropriate policies, programs and services that address health equity have a preponderant role to play in preventing diabetes complications at different (from individuals to structural) levels of change (106, 107).. Appropriate screening and intervention programs and improved access to effective health care services are required to prevent a widening of the gap in DRFC between Indigenous and non-Indigenous in Canada (86), while advocating for systemic changes to address health inequities. Indigenous peoples living in Canada are among the highest-risk populations for DRFC and screening should be carried out earlier and at more frequent intervals (12). Currently, this is not the case (108), but some studies included in the review aimed to reach a better standard of care for foot health and reduce disparities (13, 50, 76, 80, 81). Thus, establishing more healthcare services that integrate Indigenous Peoples cultures and traditions could improve access to care and the course of treatment (109). Finally, engagement is a paramount component of care for DRFC. A recent study on engagement did not identify specific data on this population (110), but it is worth bearing in mind that Indigenous populations are not less “engaged” than non-Indigenous populations (111). This is also a Western perspective on their engagement. Some populations are not difficult to reach - to mobilize - but they may find it hard to trust clinicians, researchers and policymakers (27, 112). Effects of colonialism (e.g., traumatic historical relationship with the government, health care professional too prescriptive or authoritarian, racism, discrimination, stereotypes, and structural barriers to cares) may be at the root of the heavy burden of DRFC. However, our study did not set out to precisely explore this population’s engagement, and this is an important avenue to explore in diabetic foot care.

### 4.3 Patient-oriented/centered research

It is not surprising that POR was not particularly integrated before 2020 for research with this population because we are more likely to employ Indigenous health research methodologies. In fact, actions that develop cultural safety, integrate all care spectrum and stakeholders, respect the values, customs, and traditions of Indigenous Peoples, and joint data collection to monitor progress and outcomes are a necessity in research to achieve health equity (101). There are specific methods for Indigenous population-centered research such as the use of Indigenous frameworks, western methods adapted to Indigenous context, community-based participatory research, storytelling and culture-specific methods (113). We have very little information about this in the literature reviewed, apart from community-based participatory research in recent years and the request for community permission in connection with ethical approval. Furthermore, based on our findings, it is also clear that this population needs to be more fully considered in research and health initiatives to promote culturally safe and quality health care. The predominantly western biomedical approach to health care in Canada has been identified as culturally insensitive and not inclusive of Indigenous perspectives and well-being (114). Currently, there is a lot of work being done in hospitals, but it’s a long-term effort. Patient interactions and engagement in diabetes care have been influenced by personal and collective historical experiences with health care providers and contemporary exposures to culturally inappropriate and potentially harmful healthcare (27). Moreover, social determinants of wellness are drivers of health equity and community research capacity (115).

Data regarding Indigenous people’s perspectives on foot health were scarce, yet critical. Thus, in order to develop culturally safe health care and promote positive change in foot health among First Nations people, it is imperative that stakeholders such as clinicians and researchers including Indigenous peoples perspectives (102). There is also a need to engage empowered Indigenous peoples in the foot health initiative. A recent call to action was issued to integrate traditional Indigenous and Western health models to improve outcomes as well as radical changes to reduce inequities and support the transformation of primary health care programs to empower Indigenous peoples and communities and improve chronic disease prevention and management (7, 116). As far as First Nations are concerned, they have the control and aim to achieve data sovereignty for data collection processes, and they want to own and control how that information can be used using the principles of ownership, control, access and possession, better known as OCAP^®^ (117). Therefore, Indigenous peoples are empowered to act independently and address their own health issue with research including DRFC (118, 119). POR is aligned with this and the non-Indigenous and Indigenous research community can team up for the health of all Canadians. Taking over control of health, well-being and clinical care by Indigenous peoples is a desirable way forward such as in the NUKA health project (120, 121).

### 4.4 Strengths and limitations

There are strengths and limitations to our scoping review. First, to our knowledge, this is the first comprehensive review of DRFC using a systematic method specifically targeting this Canadian population and including an equity lens and POR data. However, the high heterogeneity of the included studies makes it very difficult to obtain comprehensive results representing the situation. For this reason, we opted for a narrative synthesis and focused on studies including comparison/control to express broad trends. The chosen methodology is also Western and focuses on research done by predominantly non-Indigenous researchers, published in the Western evidence base and therefore Indigenous ways of knowing, being and acting based on their teachings and medicines are lacking. In addition, the overall quality of the evidence reported is dependent on the quality level of evidence of included studies. The use of the MMAT quality assessment tool (43) is a strength of our work as this is not mandatory with this research design but highlights the average quality of observational studies. Therefore, this is an area that needs improvement.

Second, although our database search strategy was robust and validated by an academic librarian, we may have missed data from the grey literature and specific communities. However, grey literature is rarely peer-reviewed and difficult to identify, but our attempt is a plus value to portray the overall situation. Otherwise, we minimized bias by testing our selection strategy with a two-arm independent reviewer pilot, and agreements were strong (Cohen’s kappa > 80%) (122). Due to a selection performed by two groups of reviewers, this may lead to differences in selection and extraction. We attempted to reduce this disparity by involving the lead reviewer in both arms.

Third, this review followed the recorded protocol, but was modified to improve the robustness of the methodology based on the progress of the study and evidence. This study was initiated in November 2020. The adjustment concerned the research questions on equity and POR, and the choice of the scoping design, being less restrictive, allowed this malleability. We wanted to provide a concrete analysis of the evidence regarding equity and POR to also contribute to the improvement of research in this area. Finally, although the initial research question emanated from a clinical setting dealing with Indigenous peoples and wanted to identify the overall burden of the diabetic foot disease, no patient or citizen were included in the research process as co-investigators. On the other hand, consultation with stakeholders was our way of involving them and was undoubtedly a great addition as we have conducted inclusive research, used culturally acceptable language, and discussed the results in concert with what is important for them.

### 4.5 Futures directions

With these strengths and limitations in mind, we emphasized the urgent need for robust research in Canada with Indigenous peoples, particularly integrating all factors related to equity and to consider specific socio-historical-political conditions and risk factors to worse outcomes identified in this review such as rurality/remote locations, age, sex, health care accessibility (123, 124). We highlight the catastrophic effects of limb loss on this growing population, without even considering what happens to young people (≤18 years old), but knowing that the youth is increasingly affected by diabetes (125). Indigenous peoples have different identities, cultures, and contexts in the society, but data on their specific characteristics are scarce in terms of their diversity and DRFCs. There are reportedly over 630 different First Nations communities in Canada, representing more than 50 Nations and speaking more than 50 Indigenous languages, in addition to Inuit and Métis (3). We have presented our results as one group, but each subgroup (i.e., First Nations, Métis and Inuit) need to be considered independently as they have a unique situation. Precise population definition can support a better portrayal of the situation, on the one hand, and the development of adapted interventions on the other. It is well known that user-based interventions in patient-centered research are developed and implemented more easily (126). We also strongly suggest that future studies apply national and international validated standards, recommendations and definitions for DRFC research on this population (39, 127). In addition, outcomes research needs to be more inclusive with nationally representative populations by including Indigenous peoples to better inform the national burden of this disease in Canada.

Finally, it appears from all the literature reviewed that less attention has been paid to diabetic foot disease from a preventive perspective and the major focus was related to LEAs. Although these results support previous findings (10, 86), more data are needed to better understand the burden of DFU, PAD, neuropathy, and foot deformities in Indigenous in Canada, particularly in those with additional vulnerability factors such as end-stage renal disease and/or frailty (128, 129). Researchers need to embrace Indigenous methods and co-research with two eyed seeing. The Inuit peoples of the provinces and territories, who are still poorly integrated in the knowledge of the burden of diabetic foot disease, deserves special attention in further research. While efforts have been made in recent years to identify and manage DFRCs, particularly in collaboration with the community, it is imperative that Indigenous communities and peoples be considered as partners in the promotion of quality and culturally safe health and social services for limb preservation within the research. Knowledge development with this population should move in this direction regardless of the type of study and resources and ensure adequate transfer.

## 5 Conclusion

Indigenous peoples in Canada experience a high burden of foot disease and DRFC, however since the data and high-quality studies are limited and heterogeneous, the extent of the situation may be underestimated. Even if Indigenous identity shows trends for worst health outcomes related to DRFC, it is also the synergy of socio-historical-political conditions (and colonialism) faced by Indigenous peoples that predispose them to diabetes and its complications. We have done a comprehensive review that specifically included an equity lens and POR, but this review highlights the problem of our western method. This knowledge is only the tip of the iceberg in terms of truly supporting this population through concrete and concerted action with, not for, Indigenous peoples. Social services and health care must be improved using Indigenous ways of knowing, being and acting to reach equity, especially for those living in rural and remote communities in Canada. Potential solutions lie with them. Although these results corroborate previous findings for other populations, additional data are needed to better understand the impacts of DRFC considering culture, beliefs, traditional medicine, and lifestyle of Indigenous peoples. The Indigenous peoples should be given further consideration in research and initiatives aimed at promoting culturally safe and quality health care and access. It is crucial to recognize the specific needs and prioritize prevention strategies to reduce the burden of diabetic foot disease among this at-risk population.

## Author contributions

The project was conceptualized by VB, JP, and A-ML. Data acquisition, including selection, review, and extraction was performed by VB, ST, JP, MB-F, and SL. VB performed most of the analysis with JP and ST. VB conducted the consultation. VB drafted the manuscript and was mentored and advised by DA and M-CT. All authors contributed equally to the revision of the manuscript and its final approval. All authors take full responsibility for its content.
