# Supplementary material for: Diabetic foot complications among Indigenous peoples in Canada: a scoping review through the PROGRESS-PLUS equity lens
Source: Front Endocrinol (Lausanne). 2023 Aug 14;14:1177020. doi: 10.3389/fendo.2023.1177020 (PMC10461566; doi:10.3389/fendo.2023.1177020)
Supplement: Supplementary file 1 [file DataSheet_1.docx]

Supplementary Material

Diabetic foot complications among Indigenous peoples in Canada: a scoping review through the PROGRESS-PLUS equity lens

**Virginie Blanchette^*^, Jérôme Patry, Magali Brousseau-Foley, Shweta Todkar, Solène Libier, Anne-Marie Leclerc, David G Armstrong, Marie-Claude Tremblay**

*** Correspondence:** Corresponding Author: [Virginie.Blanchette@uqtr.ca](mailto:Virginie.Blanchette@uqtr.ca)

# Search Strategy – Adapted by database but developed with MEDLINE via EBSCO.

| Concept A AND Concept B |
| --- |
| 12 OR 13 OR 14 OR 15 OR 16 OR 17 OR 18 OR 19 OR 20 OR 21 OR 22 OR 23 OR 24 OR 25 OR 26 – Concept B |
| 26. [MeSH] Indigenous Canadians OR Indigenous Peoples |
| 25. [tiab] [free text] Native Canadian and truncators |
| 24. [tiab] [free text] Metis and truncators |
| 23. [tiab] [free text] Alaskan and truncators |
| 22. [tiab] [free text] Ancestry group and truncators |
| 21. [tiab] [free text] American indian and truncators |
| 20. [tiab] [free text] first nation and truncators |
| 19. [tiab] [free text] Eskimo and truncators |
| 18. [tiab] [free text] Inuit and truncators |
| 17. [tiab] [free text] American Indian and truncators |
| 16. [tiab] Native born and truncators |
| 15. [tiab] [free text] Aborigin and truncators |
| 14. [tiab] Native and truncators |
| 13. [tiab] [free text] Indigenous |
| 12. [tiab] United States |
| 11. [tiab] Canada |
| 10. [tiab] north America |
| 1 OR 2 OR 3 OR 4 OR 5 OR 6 OR 7 OR 8 OR 9 - Concept A |
| 9. [MeSH] diabetes complications OR diabetic foot OR diabetic neuropathies OR diabetic angiopathies |
| 8. [tiab] [free text] ulcer and truncators |
| 7. [tiab] [free text] (peripheral arterial disease or peripheral artery disease or pad) and truncators |
| 6. [tiab] angiopath and truncators |
| 5. [tiab] deformit and truncators |
| 4. [tiab] [free text] amputation or amputee or amputees or limb loss and truncators |
| 3. [tiab] [free text] Neuropath and truncators |
| 2. [tiab] [free text] Foot and truncators (including pieds, feet) |
| 1. [tiab] [free text] [MeSH] Diabetes and truncators |

# Supplementary Tables 4. Details about PICO and findings

| **First author (year)** | **Sample (n)** | **Population characteristics (A-PROGRESS-PLUS)** | **Aims /interventions** | **Comparative** | **Outcomes/Major findings** |
| --- | --- | --- | --- | --- | --- |
| Chan (2021) | Approximately  18 542 persons | P: Rural, remote communities only accessible by plane or ice road  R: First nations Sioux Lookout  O: -  G: Men (51.4%) vs. Women (48.6%)  R: -  E: -  S: -  S: Program reintroduce lifestyle, activities, self-management by the community  A: 58.9% were aged older than 18 years | Evaluate the prevalence and incidence of diabetes complications. | NA | - Incidence of complications among patient with diabetes (Diabetes prevalence: 12.9%)   Surgery for leg circulation problem (e.g., LEA) incidence 0.8 (0.4-1.2). |
| Essien,  (2020) and  Essien, (2021) | 1 347 persons with LEA | P: -  R: First nation self-registered (Indian Act of Canada) in the whole province of Saskatchewan  O: -  G: 68% Male  R: -  E: -  S: Income Quantile  Highest 101 (7.5%) Vs. 620 (13.7%)  Middle 236 (17.5%) Vs. 1605 (35.5%)  Lowest 925 (68.7%) Vs. 2049 (45.3%)  Not reported 85 (6.3%) Vs. 246 (5.5%)  S: -  A: 64% aged 50 years and more  For the secondary analysis (2021) | Evaluate the incidence rate of LEA to better understand disparities. | 4520 with LEA in the general population  Population with LEA/diabetes | - Overall LEA   Diabetes (n=1098; 81.5%) vs. 2447 (54.1%) in the general population  Diabetes diagnosis: 1.7 time more likely to have a LEA for indigenous population  PAD (n=56; 4.4%) vs. 393 (16.3%) in the general population  87.7 $\pm$20.2 per 100 000 population vs. 32.4$\pm$2.3 per 100 000  Overall rate (-0.7%) in the general population vs. indigenous population rate (+4.9%)* over the 2006-2019 period   - Overall Primary LEA   869 (64.5%) vs. 3369 (74.5%) in the general population  53.6 $\pm$12.9 per 100 000 vs. 23.3 $\pm1.9 \mathrm{per} 100 000$*  Primary rate (-1.0%) in the general population vs. indigenous population rate (+5.1%)* over the 2006-2019 period   - Overall Subsequent LEA   478 (35.5%) vs. 1151 (25.5%)  34.2$\pm8.7 per 100 000 vs.$9.2 $\pm1.4 per 100 000$*  Subsequent LEA annual percent change (0.1%) in the general population vs. indigenous population rate (+4.6%)* over the 2006-2019 period     - Overall Major LEA   Annual percent change (-24.0%) in the general population vs. indigenous population (+3.3%)* over the 2006-2019 period  Indigenous are more likely to have a major LEA (adjusted RR = 1.77, 95% CI 1.23–2.54, p = 0.002)*   - Overall Minor LEA   Annual percent change (+0.8%) in the general population vs. indigenous population (+6.2%)* over the 2006-2019 period  Indigenous are more likely to have a minor LEA (adjusted RR = 1.73, 95% CI 1.28–2.34, p<0.001)*   - LEA (Age and Sex adjusted)   Age-adjusted:  153.0$\pm$17.3 per 100 000 in indigenous population vs. 31.1$\pm2.3 per 100 000 Rate$ in general population*  Higher rate for people aged 50 and over for both population  Sex adjusted:  Higher rate for male for both population  88.2 $\pm$20.5 per 100,000 in indigenous cohort vs. general population cohort (32.4 $\pm$2.3 per 100, 000)  Indigenous female almost twice likely to have a LEA that the general population (adjusted RR = 1.98, 95%CI 1.36–2.87)  Indigenous male at higher risk (adjusted RR = 1.66, 95% CI 1.30–2.13) (p = <0.001)*.   - Sub analysis (2021): Indigenous people are associated with prolonged post-operative acute care length of stay (unadjusted) OR 1.35 (1.14 – 1.60)* |
| Pace  (2020) | 2 098 persons with diabetes | P: Community characteristic (n=7) living on reserve.  R: First nation communities across 6 provinces  O: -  G: Female 55.3%  R: -  E: -  S: -  S: -  A: not known for vascular disease = 58.7 years (SD 14.2)  Known for vascular disease = 69.2 (SD 13.2)* | Identify proportion of participants meeting clinical guidelines -specified targets. (FORGE AHEAD) | Known for vascular disease (n= 376)  vs.  Unknown for vascular disease (n= 1722) | - Microvascular disease (including neuropathy, nephropathy and/or retinopathy)   87/376 = 23.1%  96/1722 – 50.6%* |
| Hayward  (2020) | 2 008 person with diabetes | P: Community characteristic (n=8)  Isolated level 87.5% (7)  Semi-isolated 12.5% (1)  Isolated 0%  R: First nation communities across 6 provinces  O: -  G: Female 57.2%; Male 42.8%  R: -  E: -  S: -  S: Total community members  500-999: 25% (2)  1000-4900:50% (4)  5000 and greater: 25% (2)  Onsite primary care physician: 37.5% (3)  A: Mean years (SD) 60.5 (14.6) | Develop quality improvement initiatives to improve diabetes type 2. | Pre-post | - Microvascular complications (nephropathy, retinopathy and neuropathy) 17.9% (359) - ORV:   Pre and Post intervention (FORGE AHEAD)  Neuropathy screening pre-intervention 10% (219) and post intervention 8.7% (175)  Adjusted for age/presence of diabetes complications) OR: 0.97 (0.76-1.23), p > 0.56 |
| Shah (2019) | 2002/03 cohort^a^ : 13 573  2014/15 cohort^b^ : 23 011 | P: Urban ^a^23.8%  ^b^27.3%  Semiurban ^a^20.5%  ^b^19.9%  Rural ^a^18.6%  ^b^16.8%  Remote/missing ^a^37.1%  ^b^36.0%  R: FN identified under Indian act of Canada  O: -  G: Male ^a^44.8% ^b^46.4%  Female ^a^55.2% ^b^53.6%  R: -  E: -  S: -  S: -  A (years) ≤ 19 ^a^1.3% ^b^1.3%  20-34 ^a^10.3% ^b^7.0%  35-49 ^a^31.1% ^b^24.6%  50-64 ^a^37.2% ^b^40.1%  65-74 ^a^14.1% ^b^1.3%  75-105 ^a^6.0% ^b^9.2% | Evaluate the revascularization/  LEAs. | 2002/03 cohort^a^ :  n=680 880  non FN  2014/15 cohort^b^ :  n=1 364 136  non-FN | - PAD: revascularization rate comparable. Results suggest that PAD may be underdiagnosed or undertreated among FN people in Ontario. - LEA: 50% minor LEAs and 50% major LEAs   LEAs frequency 3-5 times higher to comparative (across sex, age and location), ≤ 44 years: LEA 6 times more frequently for FN rate higher for male, rate increasing with rurality and age  Living in remote community = high-risk for LEA   - Mortality after LEA: 15% higher in FN compared with non-FN   Adjusted hazard ratio 1.15, 95% CI [1.05–1.26], with median survival of 3.5 years for FN versus 4.1 years   - ORV: This disparity may be driven in part by poor access to health care, particularly specialized services for wound care and rehabilitation. One of the health care barriers: living in remote communities. |
| Loewen (2017) | 1 585 are adults with diabetes (representing 11% of the total adult population) | P: -  R: FN Sioux Lookout Mena Ya Win Health Centre area in northwestern Ontario  O: -  G: Male/female ratio who underwent LEA 75/25  R: -  E: -  S: -  S: -  A: 50.2 years ± SD 8.7 | Evaluate diabetes and LEA on 4-years period. | n total population = 22 776 (85% FN)  LEA Compared to Central Toronto: 1.81  North East Ontario 1.45  North West Ontario 3.13  Age Ratio: 74/26 | - LEA: 1 078.5 per 100 000 for FN vs. 146.5 per 100 0000 for comparator (7 times the provincial rate) - Rate of BKA per 1000 adults’ patients with DM2 = 5.68 so 3-time higher than other comparators - Average age BKA with type 2 diabetes = 50.2 yr SD 8.7 (FN) VS 64.0 yr SD 2.3 for the 3 comparators - Sex ratio similar between FN and comparator - Development of DFU management protocol |
| Turin (2016) | 70 631 persons | P: Rural 47.7%  Urban 53.3%  Unknown 0.0%  R: Albertan FN communities including Blackfoot, Cree, Chipewyan, Dene, Sarcee and Stoney; Albertan Health Registry file registered Indigenous Affairs and Northern Development Canada  O: -  G: 49.9 % female  R: -  E: -  S: Income quintile  1 (lowest): 42.5%  2: 14.7%  3: 10.5%  4: 7.9%  5: (highest) 9.4%  Unknown: 15.1%  S: -  A: 34.4 years ± 12.6 | Evaluate the lifetime risk of diabetes and estimate the disease burden. | n = 2 732 214  A (years): 40.7 ± SD 16.3  G: 49.5% female  Baseline peripheral vascular disease = 0.6% | - Baseline peripheral vascular disease 0.2% - The lifetime risk of developing diabetes is greater in the FN population than in the non-FN population. |
| Al Sayah (2015) | 48 persons | No information related to indigenous population only with diabetes and foot outcomes. But aggregate data were provided regarding sex, ethnicity, education level, income, dwelling, health literacy, age. | Evaluate the prevalence and predictors of foot disease, self-care and clinical monitoring. | White Caucasian:  n = 1 852  Others: n = 140 | - Neuropathy: - 10 (20.0%) vs. 340 (18.4%) white Caucasians vs. 22 (15.7%) others - Peripheral Vasculopathy: - 11 (22.9%) vs. 516 (27.9%) white Caucasians VS 30 (27.9%) others - Foot or leg ulcers or infection: - 3 (6.3%) vs. 105 (5.7%) white Caucasians VS 11 (7.9%) others - Gangrene or LEA: - 0 vs. 23 (1.2%) white Caucasians vs. 5 (3.6%) others |
| Maple-Brown (2012) | 188 persons | P: -  R: Remote Canadian Group (Group 3) located in Sandy Lake, Ontario (subarctic boreal forest region), with a population of 2300 Oji-Cree.  O: -  G: Female n=113 (60%)  Other characteristics see Hanley, 2005)  R: -  E: -  S: -  S: -  A: 47 years ± 13 | Evaluate diabetes complications in different Indigenous population in Australia and Canada. | Other Australian Indigenous  Remote Australian study group (Group 1) n=37  A (years): 51 ± 8  G: Female n= 20 (54%)  Urban Australian study group (Group 2)  n = 99  A(years): 53 ± 10  G: Female n=75 (76%)  Remote Australian:  Peripheral neuropathy: 10 (28%) (total n= 32)  Abnormal 10g monofilament:7 (19%)  PVD: 2 (5.4%)  Current DFU: 1 (3%)  LEA: 0  Urban Australian:  Peripheral neuropathy: 9 (9%) (n=82)  Abnormal 10g monofilament: 8 (8%)  PVD: 12 (12%)  Current DFU: 6 (6%)  LEA: 2 (2%) | Peripheral neuropathy:  68 (47%) (total n =146)* (significant with the comparative groups  Abnormal 10 monofilament:  22 (15%)  Peripheral vascular disease:  16 (11%) (total n = 145)  Current DFU:  7 (5%) (total n= 142)  LEA:  0 total amputation (total n= 144)  NOTE : We identified confusion regarding the neuropathy and PAD data reported in the table of this study. |
| Martens (2010 updated in 2012); 2002 | 5 846 persons | No information related to indigenous people with DM only  P: -  R: Métis from registries and self-reported in all Manitoban communities  O: -  G: -  R: -  E: -  S: -  S: - | Health and health care utilization | Other people from MB  n = 1 148 401 | - Crude Prevalence Métis with LEA =1.56% Métis - Total of 135 LEA - LEAs rates for Métis: 24.1 vs. 16.2 per 1000; RR 1.49; 49% higher compared to others MB* - Both Rural south (22.8 VS 16.0 per 1 000) and Mid (28.3 VS 22.3 per 1 000 showed trends toward higher rates for Metis compared to others - Norther is the opposite trend (27.5 vs. 36.4 per 1000); this is explicated by the FN in the north part of MB that have a high rate of LEA due to diabetes - Metis are a similar risk of LEA compared to other Manitoban after controlling sex, age, income, geographic area, mental and physical comorbidities, continuity of care - Risk of LEA is higher: male (OR 1.94, 95% IC 1.71-2.21; p<0.001); living neighborhood income area and for those with comorbidities, older - See the same physician for at least one-haft or their visit over 2-year period) was associated with lowest risk of LEA (OR 0.71, 95% IC 0.62-0.81; p<0.001) - High risk of LEA in Metis male VS female (OR 2.36, 95% IC 1.50-3.71; p<0.001) - Continuity of care associated with lower risk of LEA in Metis (OR 0.62, 95% CI 0.40-0.96; p<0.04)   2002: LEA rate 6.2/ 1000, twice other first nation of the province (3.1 / 1000) and 30X non-first nations without diabetes |
| Reda (2012)  For diabetic:  Previous study data (McItyre 2007) | 27 Indigenous with end-stage renal disease (ESRD) and diabetes (41%)  and  39 non-indigenous with ESRD and diabetes (59%) | No information related to indigenous people with DM only.  P: Sandy Lake, 2000 Km Northwest Ontario/ remote  R: Oji-Cree  O: -  G: -  R: -  E: -  S: -  S: Nursing station and 2300 inhabitants  Disability:8 ESRD patients on hemodialysis (46 non-diabetic + 66 diabetic patients + 11 UNKNOWN) | Effects of a preventive program related to diabetic foot complications. | All patients without diabetes representing (Indigenous and Non-indigenous): n=46 (37%) | - Data including indigenous and non-indigenous - In the diabetic group: - PAD: 11/66 (17%) - Neuropathy: 30/58 (52%)* (with the current study) - Tibia Brachial Index <0.7 : 23/60 (38%) - Absent pedal pulse: 21/58 (36%)* (with the current study) - LEA: 9/58 (16%) - Current DFU: 9/58 (16%) - Charcot Foot: 5/58 (9%) |
| Harris (2011) | 885 persons | P: isolated community, non-isolated, and remote-isolated/semi-isolated  R: FN from 19 communities >500 habitants O: -  G: Female 63.3%  R: -  E: -  S: -  S: -  A (years): 54.9 SD 14.2 | Document the clinical management of  type 2 diabetes and related complications in Canada’s FN. | NA  Compared to a  general population (n = 2493)  Neuropathy: 8%  Diabetic foot disease: 2.2%  PAD: 6%  Amputation: 1.0% | - Neuropathy: 96/885 (10.8%)   Across 19 communities = 0-40%   - Diabetic Foot disease: 47/885 (5.5%)   Across 19 communities = 0-34%   - PAD: 22/885 (2.4%)   Across 19 communities = 0-13.7%   - Amputation (type not mentioned): 14/885 (1.6%) - Across 19 communities = 0-6% |
| Oster (2010)  Data related to Oster 2009 | 2 102 persons | P: 43 communities on-reserve  R: 62%of DM cases in AB FN, Cree speaking  O: -  G: 62.5% female  R: -  E: -  S: -  S: -  A (years): Male 54 ± 13.9  Female 53.4 ± 14.6 | Evaluation of screening program to reduce the burden of diabetes. | Longitudinal follow-up (6 yr) compared to the baseline. | ORV: Foot risk category (0 = no abnormality; 1 = sensory loss, low  risk; 2 = sensory loss and presence/history of deformity, moderate risk; 3 = presence/history of plantar ulcers, high risk)   - % with no abnormality (0): Male 63.0%* (59.5-66.5) Female 71.3%* (68.7-73.9) - % at high risk (3) Male 7.7% (5.8-9.7) Female 5.2% (4.0-6.5) - Foot abnormalities more prevalence in male (p<0.05) |
| Shah (2010);2011 | 13 173  Métis | Metis diabetes prevalence based on standardized age and sex: 8.13/100 people (compared with 6.45 for the general population)  P: Metis nation of ON citizens registry  R: 20% Métis population of ON  O: -  G: estimated  R: -  E: -  S: estimated; Metis nation was poorer than the general Ontario population  S: -  A: estimated | Describe diabetes among Metis and look to the type of treatment received. | General population of Ontario | - LEA   Standardized age et sex: Metis: 0.10/100 people with diabetes compared to 0.16/100 in the general population with diabetes (difference was not statistically significant)  Procedure to reopen blood vessels in people with diabetes: Metis 1.30/100 people with diabetes (standardized)  Gen Pop : 0.81/100 people with diabetes (standardized) (Difference is not statistically significant) |
| Lovell (2009) | 8 persons (1.6% of the total n = 501) | May be not associated to diabetes  No information related to indigenous people with DM only  P:  R: 8% Indigenous  O:  G: yes  R:  E: yes  S: yes  S:  Age: yes | To assess public knowledge of PAD in Canada. | Other Canadians | - FN were less represented in the overall study - Authors supported that Indigenous identity is an important factor with health disparity in Canada also for PAD. |
| Oster (2009) | 743 self-referred AND completed survey + screening FN with diabetes | P: 43 communities on-reserve  R: 60% FN on-reserve in AB  O: -  G: Male n=254; Female n=489  R: -  E: -  S: -  S: -  A (years): mean 53 | Describe the state of diabetes care among FN individuals with diabetes living on reserve. | Self-reported data to clinical examination comparison for undiagnosed foot complications | Foot risk category (0 = no abnormality; 1 = sensory loss, low risk; 2 = sensory loss and presence/history of deformity, moderate risk; 3 = presence/history of plantar ulcer, high risk)   - No abnormality: 63%, 95% CI (59.5-66.3) - Low risk = neuropathy: 15%, 95% CI (9.5-20.7) - Moderate risk: 16%, 95% CI (10.6-21.8) - High risk: 6%, 95% CI (0.5-10.9) - More male at high risk of foot complications (p <005)* - 11% undiagnosed foot complications (5.2-16.8%) - 21% had their feet examined by a podiatrist within the previous year - 39% had ulcers or sores on their feet or legs that would not heal for longer than 1 month |
| Bruce (2008) | 483 FN | P: Sandy Bay in MB, on-reserve  R: FN speaking local language Saulteaux  Yes Male 204 (89.5% of males)  Female 204 (83% of females)  No Male 24 (10.5%)  Female 41 (17%)  O: Employment status  Employed Male 60 (26% of males)  Female 77 (31% of females)  Not employed Male 169 (74%)  Female 170 (69%)  G: Male 48% (230)  Female 52% (253)  N total = 483  R: NA  E:  Grade 9 or +: Male 89 (39%)  Female 131 (54%)  < Grade 9: Male 138 (61%)  Female 111(46%)  S: -  S: 200km from Winnipeg, nearest major urban center and accessible by road year-round  Age (years): Male 37.3 ± 12.5  Female 38.2 ± 12.1 | Determine the prevalence of and risk factors  for diabetic neuropathy in a Canadian FN population. | NA | - Neuropathy: n =34/483 (7%) - Pain: n = 38 - Numbless/tingling: n = 34 - Previous DFU: n = 1 - Previous toe amputation: n = 3 - Neuropathy was found in 5% of normoglycemic patients, in 8% of patients with a new IFG or a new diabetes and in 15% of patients with a previous diagnosis of diabetes. - The odds of having neuropathy were 2.7 times greater for women than for men and 3 times greater among those who completed less than grade 9 compared with those who completed grade 9 or higher. - The odds of neuropathy increase for each percent increase in A1C; the risk for neuropathy is twice as great for someone with an A1C of 9 versus 6%. - Neuropathy risk is three times greater for someone who smoked 30 pack-years than for someone who smoked 10 pack-years. - The risk of neuropathy is twice as great for participants with a homocysteine level of 9 μmol/l versus those with a level of 13 μmol/l (hyperhomocysteinemia defined as >12 μmol/l) |
| Dannenbaum  (2008) | 1363 Cree with a diagnosed diabetes type 1 or 2 | P: Cree of Eeyou Istchee live in 9 remote communities in a vast region of boreal forest in Northern QC  R: Historically, Cree life consisted of semi-nomadic  bush living centered on hunting and trapping.  O: -  G: female 62.3%  R:-  E: -  S: -  S: 5 communities are situated on the Eastern shores of James Bay and Hudson Bay, and 4 communities lie inland along the main waterways, close to the city of Chibougamau, QC  A (years):  10-19 n =9  20-29 n = 88  30-39 n =276  40-49 n =293  50-59 n = 319  60-69 n =241  ≥70 n = 138 | Overview of the epidemiology and clinical  management of diabetes. | NA | - Neuropathy: 109(8% of the total population of 1363 with a diagnosis of diabetes) - DFU (chart): 12 (0.9%) - LEA (chart): 9 (0.7%) - Peripheral arterial disease (chart): 26 (1.9%) |
| Attawar (2006) | 102 (out of 486 participants with disglycemia) with DFU problems and; 9 for interviews; 31 screening | P: FN in MB  R: Most residents are fluent in English Saulteaux, an Ojibway dialect is  also commonly spoken by community members  O: Employment status  Employed 22% (22)  Unemployed 78% (80)  G:  Male 44% (45)  Female 56% (57)  R: Although, the community remains predominantly Roman Catholic, many people retain aboriginal spiritual beliefs and practices. Traditional healers live and practice in the community.  E: -  0 grade 6: 40% (41)  Grade 7-12: 38% (39)  Post-secondary:21% (21)  S: In 2003, almost 39% of  community residents received social assistance as their sole income  S: The community is located in southern MB, 90 km from the nearest  rural town, and is accessible year-round by road  Age (years): 20 to 69 | Gain better understanding of why FN communities are more vulnerable to diabetes foot complications and amputation, a case study about foot care was conduct. | NA | - Community (2003 = 3000 habitants): higher prevalence of diabetes related LEA (6.2 per 1000) = 16X relative to all MB - Registered cases of LEA in the community: - 1990 = 14   1997 = 12  2001 = 7  2002-2003 = 8   - 47/102 major signs and symptoms for foot complications:   Vascular screening participants (n = 31)  History of DFU: 32% (10/31)  History of LEA: 10% (3)  LOPS: 64% (20)  Skin problems: 58% (18)  Nail problems: 32% (10)  Foot deformity: 16% (5)  Current DFU: 10% (3)  Doppler  Mild claudication: 16% (5)  Incompressible vessel: 19% (6)   - Interview results for patients and their caregivers (n =9)   Several people reported experiencing chronic,  persisting foot pain.  Sensory loss appeared to be a common source  of serious injuries among amputees.  Impact of LEA: For several individuals,  amputation was life altering as it had restricted  their ability to participate meaningfully in the  community. |
| Virani (2006) | 1 151 patients with known diabetes but only 274 evaluated for limb complications | P: All 44 first nations communities  R: Cree speaking  O: -  G: Female 64% (741); Male 36%  R: -  E: -  S: -  S: - Community-based care, care trajectory with the urban Centre; Social worker in the community  Mean Age (years): 54 | Identify diabetic foot complications and evaluate the impact of a mobile screening clinics on complications, educations and satisfaction. | Pre and post intervention | - Limb complications screen (0: no abnormality; 1: neuropathy; 2: neuropathy and history of ulcer; 3: presence or history of ulcer) not precise.   Mean at baseline (limb complications screen): 0.54  Mean at follow-up (6-12 months) post intervention: 0.50; p>0.5 |
| Martens (2007) | 3 582 with diabetes on 23 844 FN | Age and sex standardized diabetes prevalence per 1000  ^1^Keewatin Tribal Council: 149*  ^2^Independent First Nations North 173*  ^3^Swampy Cree Tribal Council 205  ^4^Island Lake Tribal Council 220  ^5^South-East Resource  Development Council 219  ^6^West Region Tribal Council 231*  ^7^Interlake Reserves Tribal Council 207  ^8^Independent First Nations South 234*  ^9^Dakota Ojibway Tribal Council 249*  P: 9 FN communities in MB study  R: On-reserve  O: -  G: Adjusted but not detailed  R: -  E: -  S: Average household income ($)  ^1^ 30 730  ^2^ 31 442  ^3^ 29 891  ^4^ 28 132  ^5^ 24 428  ^6^ 22 607  ^7^ 22 751  ^8^ 23 719  ^9^ 20 503  S: 39% FN living North MB  The northern part of the province has many remote communities  accessible only by airplane or winter road.  Age (years): 20-70 | Describe the prevalence of diabetes and  the rates of LEA with diabetes comorbidity; determine ecologic association of diabetes indicators with measure income, access to specialist and geography. | Overall population of Manitoba. | - LEA: 3.39 FN compared to 0.19 /1000 for all MB (adjusted rate) (represented 8X all Manitobans) - LEA rate varied by tribal council (1.19 to 6.16 per 1000) - Associated health care access (consultation rates)   Age- and sex standardized annual LEA rates per 1000  ^1^ 1.37  ^2^ 2.20  ^3^ 5.03  ^4^ 3.77  ^5^ 5.07  ^6^ 3.85  ^7^ 3.95  ^8^ 1.19  ^9^ 6.16*  LEA rate inversely proportional to specialist access   - 234 LEA on total 116 071 FN registered on 5-year period (crude unadjusted rate) |
| McIntyre (2007) | 64 Indigenous on hemodialysis /127  (and 63 non-indigenous) | P: MB who received hemodialysis at the Health Science Centre, a major tertiary care referral hospital  R: self-identified as aboriginal  O: Mean year employed (n = 22)  19 ± 9  G: Male 35(55%); Female 29 (45%)  R: -  E: -  S: -  S: -  Age (years): 56 ± 11 (not available for 2 patients) | Profile of lower extremity morbidity in diabetic patients with end-stage renal disease. | Non-indigenous  (n = 63)  Caucasian  44 (35%)  Filipino 11(9%)  Asian  5 (4%)  East Indians  2 (1.5%)  Black  1 (0.5%) | - Sensory neuropathy   34/36 (94%) vs. 30/37 (81%)   - Absent vibration   20/28 (71%) vs. 24/35 (69%)   - Unable to occlude vessel   Left: 14/19 (74%) vs. 3/19 (16%)  Right: 12/19 (63% vs. 12/18 (67%)   - Ankle brachial Index < 0.9   Left: 1/19 (5%) vs. 3/19 (16%)  Right: 1/19 (5%) vs. 5/18 (27.8%)   - Ankle brachial index 0.9 to 1.4   Left: 6/19 (32%) vs. 6/19 (32%)  Right: 4/19 (21%) vs. 3/18 (2%)   - Absent pulse   Dorsalis Pedis: 10/36 (28%) vs. 3/41 (7%) * 0.02  Posterior Tibialis: 5/34 (15%) vs. 4/40 (10%)   - Poor nail conditions   24/32 (75%) vs. 25/37 (68%)   - Hairless toe   22/34 (65%) vs. 22/37 (59%)   - Dry skin   22/34 (65%) vs. 21/36 (58%)   - Prior DFU   47/63 (75%) vs. 26/63 (41%) *0.0002   - Current DFU   11/37(30%) vs. 10/37 (27%)  Mean number of DFU 2.1 ± 2.0 1.2 ± 2.1;  p<0.05*   - Charcot foot   14/62 (23%) vs. 2/6 (30%); p =0.01*   - Prior Osteomyelitis   22/60 (37%) vs. 7/64 (11%); p = 0.0008*   - LEA 22/61 (36%) VS 11/63 (17%); p<0.05* - Reason for inadequate foot care:   Financial cost, lack of family support, language  barrier  All the cohort (Indigenous and non-indigenous)  Risk factors for mortality:  Mean number of prior foot ulcers, the proportion of patients with either an absent dorsalis pedis pulse, prior myocardial infarction, LEA, prior angiogram, not performing a daily foot inspection, occluded vessel detected by angiography. |
| Rose  (2007) | 101 Indigenous with diabetes /325 patients with diabetes | P: MB  R:  Urban:38(38%)*  Rural:1(1%)*  Reserve:62 (61%)*  O: Employment  Employed: 16 (16%)*  Retired: 37 (36.5%)*  Social assistance: 45 (44.5%)*  Not available: 3 (3%)  G: Male 58 (57%); Female 43(43%)  R: -  E: -  S: -  S: -  Age (years): 55 ±13 | Evaluate the clinical outcomes in Canadian non-  Indigenous and Indigenous patients with diabetes and DFU managed at a multidisciplinary, tertiary care diabetic foot clinic. | Non-indigenous  (n = 224)  218 (97%) were Caucasians | - No baseline characteristics only for the indigenous population. - Indigenous patients had a shorter average time from initial clinic visits to major LEA   (50 ± 64 weeks VS 62 ± 56 weeks; p < 0.01)*   - Residence in a rural or on-reserve community   also correlated with shorter average time from initial clinic visit to major LEA (rural or reserve, 45 ±56 weeks; urban, 66 ±61 weeks; p < 0.002)*.   - LEAs :   Total : Indigenous 24 (24%) vs. 34 (15%)  Toe: 12 (39) vs. 22 (53)  Forefoot: 4 (13) vs. 3 (7)  Below knee: 14 (45) vs. 14 (33)  Above knee: 1 (3) vs. 3 (7)   - Survival time without major LEA (defined as proximal to the toes)   Indigenous ethnicity (p<0.01)*  With factors = 167 weeks ± SE 10 (101)  No risk factor = 202 ± SE 6 (224)   - Risk factor for poor outcomes with DFU   Indigenous ethnicity is NOT statistically significant  Bad outcomes defined as static, progression, LEA, death  Good outcomes defined as healed of healing DFU   - Good outcomes   Risk factor 53 (28%); no risk factor 137 (72%)   - Bad outcomes   Risk factor 48 (36%); Poor outcomes 87 (64%)   - Indigenous patients with a DFU had a LEA approximately 12 weeks earlier than in non-indigenous patient with a DFU. |
| Goulet (2006) | 84 Indigenous patients  and 77 have (92%) diabetes | P: FN/M/I  identified from their treaty numbers or from references made to ethnicity in the medical record.  R: -  O: -  G: -  R: -  E: -  S: -  S: -  Age (years) at bypass: 64 ± 11* | Evaluate the outcomes of revascularization for peripheral arterial disease | Non-indigenous and diabetes (n = 249)  Total of 594 non-indigenous patients with PAD that required a revascularization | - 92% of Indigenous patients with PAD requiring revascularization with bypass, had diabetes compared to 42% in the subgroup of non-indigenous patients that were diabetics* p<0.0001 - Indicative for surgery (irrespective of the presence or absence of diabetes):   Rest pain: 43 (40%) vs. 401 (56%); <0.002  Claudication: 9(8%) vs. 206 (29%); p<0.0001  Gangrene: 63 (58%) vs. 112(16%); p<0.0001  Non-healing UPD: 29 (27% VS. 131 (18%); p<0.05  Acute ischemia: 7(6%) VS. 59 (8%); NS   - Total of 828 procedures: 108 on Indigenous and 720 on non-indigenous - LEA (at or below knee level)   At least one toe: 35 (32%) vs. 54(8%)  Forefoot: 6 (6%) vs. 2 (0.3%)   - Complications after revascularization procedure (non-significant)   Limb loss: 2(2%) vs. 4(1%)  Wound infection: 4 (4%) vs. 25(3%)  Death: 2 (2%) vs. 20 (33%) |
| Reid (2006) | 169;  168 Indigenous with diabetes type 2 and 1 with diabetes type 1 | P: FN and M people from diverse tribal, cultural and political identities; “treaty-status”  referred to the status of an Indigenous person who was a  member of a band that had signed a treaty with the government of Canada  R: Remote community in Northern MB, Cree and English Languages  O: -  G: Male 68 (40%); Female 101 (60%)  R: -  E: -  S: -  S: Community setting of Norway House with on-site staff  A (years): Mean 56 ± 12 | Determine the prevalence of foot  complications, ongoing foot care, and footwear use in people with diabetes. | NA | - Monofilament - 10/10: 103 (61) 95% CI [54.0 to 69.0] - 7-9/10: 26 (15) [9.3 to 20.3] - 0-6/10: 40 (24) [17.2 to 30.2] - Vibration sensation - (+) Hallux, (+)Lateral Malleolus 118 (70) [62.7 to 76.9] - (−)H, (+)LM 18 (11) [5.9 to 15.5] - (−) H, (−)LM 19 (11) [6.3 to 16.1] - (+) H, (−)LM 9 (5) [1.9 to 8.7] - (−) H, unknown LM 4 (2) [0.0 to 4.8] - Proprioception (hallux) - Present 126 (74) [67.9 to 81.3] - Absent 41 (24) [18.0 to 31.2] - Unknown 2 (2) [0.0 to 3.6] - Pinprick sensation - Present 134 (79) [73.1 to 85.5] - Part of foot present 23 (14) [8.3 to 18.9] - Absent at or above ankle 12 (7) [3.1 to 11.1] - Pulse - All present 99 (59) [51.6 to 66.8] - DP/ PT absent 49 (29) [22.0 to 36.0] - No foot pulse 18 (11) [5.5 to 14.7] - Pulse present only at graft 2 (1) [0.0 to 2.9] - Unknown 1 (1) [0.0 to 1.8]   Foot and Ankle complications: Number of complications totalizing 418: number of complications / Number of persons   - Toenail pathology 102 (24%) / 102 (60%) - Deformity: 98 (23%) / 86 (51%) - Hallux valgus 52 (12%) / 52 (31%) - Claw toe 21 (5%) / 21 (12%) - Hallux rigidus 11 (3%) / 11 (7%) - Flatfoot 5 (1%) / 5 (3%) - Cavus foot 3 (0.7%) / 3 (2%) - Long second toe 3 (0.7%) / 3 (2%) - Ankle deformity § 1 (0.2%) / 1 (0.6%) - Heel pad atrophy 1 (0.2%) / 1 (0.6%) - Dorsal exostosis 1 (0.2%) / 1 (0.6%) - Callus 77 (18.4%) / 77 (46%) - Neuropathy: 40 (10%) / 40 (24%) - Past or present DFU: 25 (6%) / 25 (15%) - LEA 6 (1.4%) / 5 (3%) - Toe: 3 (0.7%) / 3 (2%) - Transmetatarsal: 1 (0.2%) / 1 (0.6%) - Transtibial: 2 (0.5%) / 2 (1%) - Charcot arthropathy: 1 (0.2%) / 1 (0.6%)   Summary: There were 139 (82%) individuals  who had 418 diabetic foot complications (average, 3.0 complications per subject with complications), including toenail pathology, foot and ankle deformities, calluses, impaired pulses, neuropathy, past or present DFU, LEA, and Charcot arthropathy.  Risk classification showed that 69 (41%) individuals  were at risk for future DFU.  In a 7-year period, foot problems accounted for 498 local emergency visits (18%);356 (16%) hospitalization, 109 (11%) non-emergency transfer; 4 (6%) emergency transfer to a tertiary hospital |
| Hanley (2005) | 189 persons | P: Sandy Lake FN  R: Remote community, on reserve  O: -  G: Female 65%  R: -  E: -  S: -  S: -  Age (years): 46.5 ± 13.3 | Determine  the prevalence  of micro- and macrovascular  complications among Indigenous Canadians  who have type 2 diabetes and to identify risk factors that are associated with these conditions. | NA | - Neuropathy: n =147, 46.3% (38.1-55.6) - PAD: - Ankle Brachial Index <0.95   n=141; 14.9% (9.7-22.1)   - Claudication   n=183; 6.0% (3.2–10.8) |
| Meatherall (2005) | 21 Indigenous underwent LEA, not receiving dialysis | P: MB or Northwestern ON FN and Métis; Urban are resident of Winnipeg  Urban: 8  Rural: 13; communities of less than 20 000 habitants  R: English as primary language  Urban 4 (50%); rural 2 (15%)  O: Employment status  Working: urban 1 (13%); rural 4 (31%)  Retired/pension: urban 2 (25%); 4 (31%)  Not working urban 5(63%);5 (38%)  G:  Urban: 7 males; 1 female  Rural: 8 males and 5 females  R: -  E:  No formal education: urban 0(0); rural 7 (54%)  Grade 1-6: urban 1 (13%); rural2 (15%)  Grade: 7-12: urban 5(63%); rural4 (31%)  Postsecondary: urban 2 (25%); rural 0 (0)  S: -  S: -  A (years): 61 ±7l  Disability: not receiving dialysis. | Compare and contrast disability and quality of  life in Indigenous and non-Indigenous people with  diabetes who had LEA. | Non-indigenous  (n =23; 16 from urban area and 7 from rural area) who underwent LEA, not receiving dialysis. | - LEA on indigenous people: younger and first major LEA before non-indigenous - Mean age first LEA: 57 ± 7 years vs. 64 ± 11 years* (p<0.015) - Current LEA level   Unilateral LEA (n =34)   - Syme: 1 vs. 0 - Below knee: 11 vs. 12 - At knee: 2 vs. 2 - Above knee: 1 vs. 5   Bilateral LEA (n= 10)   - Bellow knee/below knee: 5 vs. 4 - Above knee/above knee: 1 vs. 0   Status of intact leg (n = 34)   - Poor circulation: 10/15 vs. 10/19 - Muscle cramps when walking: 6/15 vs. 4/19 - Constant leg pain:1/15 vs. 1/19 - Wounds /ulcers: 4/15 vs. 4/19   Phantom limb pain: 6/21 (29% vs. 14/23 (61%); p <0.032*   - Wounds: 3/21 vs. 1/23 - Qualitative data: no difference about both groups - 19/37 (51%) feel distress about LEA - 8/37 (22%) please because pain relief - Reaction to LEA = acceptance 21/34; 62% more frequent in urban that rural - 9/34 sense of guilt or regret after LEA - 27/40 expressed satisfaction at the time of the interview - Participants with DFU and foot deformities for years feeling ready for LEA - 7/10 rural indigenous stated that will not walk again - Several people described feelings of regret, self-blame, and guilt regarding their general health, diabetes, and LEA. - Urban non-Indigenous subjects received a prosthesis more frequently than urban Indigenous subjects, consistent with the longer mean duration of rehabilitation after LEA in the former group - The rural non-Indigenous people had the greatest frequency of prosthesis use both inside and outside the home for all activities and the least frequent use of an ambulation aid outside the home. - Rural Aboriginal subjects had markedly greater incidence of several comorbidities. |
| Pollex (2005) | 189 ;  173 with available DNA and 138 with vascular test and genotyping | P: Sandy Lake, Ontario, is a remote Oji-Cree community, found at the 55th parallel of latitude, in the  subarctic boreal forest of central Canada.  R: FN  O: -  G: Male 20 (35%) with PAD and 118 (35.6%) with no PAD  R: -  E: -  S: -  S: -  A (years): 48.0 ±11.2 | Investigate a possible role for the MTHFR 677C>T  gene polymorphism with PAD in subjects with type 2 diabetes from an isolated Indigenous Canadian population | Indigenous population with no gene and no PAD | - Presence of intermittent claudication - PAD: 14.5% - No PAD: 85.5% - Association between gene 677c<t SNP with PAD*; Significantly more individuals carrying the 677T allele had PAD, as diagnosed by Ankle brachial Index (26.7% vs 11.1%, P =0.042); particularly when measured on the right side (23.3% vs 8.33%, P = 0.047)* - No significant association between MTHFR genotype and intermittent claudication, a much more advanced stage of PAD - MTHFR 677T carriers had an increased risk of PAD [OR 3.54 (1.01, 12.4), P = 0.049* |
| Iwasaki (2004) | 26 persons | P: -  R: M and FN  O: Unemployed: 18 (69%); 8/8 FN female, 6/9 FN men, 4/9 M female  Full-time employment: 3 M female  Part time: 3 FN male  Casually employed: 1 FN male  G: 17 females; 9 males  R: -  E:  University degree:1  Completes Grade 12: 6  Less than Grade 12: 16  Not reported: 4  S: Household income  Less than 20 000$: 198 (69%)  S: -  Age (years): 26 to 69; mean 43.9 | Gain an understanding of the  nature of stress among Canadian Indigenous women and men living with diabetes. | NA | - Related to LEA: - Fear about the future (self and family); - I have had diabetes for 30 years, and stress, well, it’s to the point where I’m getting limited to do what I want, and I realize that if I live another 20 years, I may not be able to have the legs to walk with or the eyes. - Suffering the complication if diabetes stress resulting from complications related to/or caused by diabetes including kidney removal, an enlarged liver, loss of a leg, and loss of sight. - “My husband lost his leg to diabetes, and he lost his eyes—he’s going blind.” (Métis female) |
| Légaré (2004) | 1076 persons with diabetes type 1, 2 and gestational diabetes/glucose intolerance | No information related to indigenous people with DM and LEA only  P: On reserve, remote community  R: Cree of Eeyou Istchee, Baie James, Qc, FN  O: -  G: Male 404 (84.9%); females 672 (70.2)  R: -  E: -  S: -  S: -  A: yes, but not detailed | Estimate the proportion of disease complications associates with diabetes, hospitalization; validate the database. | NA | - LEA: 7 hospitalizations; total of 5 cases (4 females and 3 males)   NOTE: not a lot in 5 years, limits of the database; only hospitalization with major amputations, no minor amputations   - 6 toe amputations and 1 leg amputation - Age group for LEA: - 40-59 years = 3 hospitalizations - 70 and over = 4 hospitalizations - Total hospitalizations (associated with these LEAs) = 81 days |
| Thommasen (2004) | 70 persons with diabetes type 2 | No information related to indigenous people with diabetes only  P: Bella Coola Valley, isolated and rural communities located in coast region of BC; Stuie and Atnarko  R: 46% of people are Indigenous descent, a part of traditional territory of the Nuxalk Nation, a tribe of Salish-speaking Coastal Indians  O: -  G: -  R: -  E: -  S: -  S: - | Identify which medical disorders are significantly associated with the population with diabetes in an isolated, rural community, and determine the difference between indigenous and non-indigenous population. | Non Indigenous with type 2 Diabetes  (n= 56) | - PAD: 5 (7.1%) vs. 4 (7.1%); NS - Neuropathy: 7 (10.0%) vs. 6 (10.7%); NS |
| Jin (2002) | 927 Indigenous with diabetes | No information related to indigenous people with diabetes only  P: -  R: -  O: -  G: -  R: -  E: -  S: -  S: -  A: yes, but not detailed (35-80 years) | Describe hospitalization rate for diabetes and its complications. | Population (with diabetes) but with no indigenous status (16 920) | Unadjusted crude rates:   - Neuropathy and amyotrophy:   4 (1/100 000 person years) vs. 124 (1/100 000 person years)   - Other atherosclerosis (including gangrene, other peripheral arterial disease):   22 (4/100 000) vs. 1 384 (8/100 000) |
| Hernandez (1999) | 10 persons | P: FN community Southwestern ON  R: unknown but able to understand and speaking English  O: NA  G: 6 females;4 males  R: NA  E: NA  S: NA  S: NA  Age (years): 32 to 75 | Investigate the experience of  Type 2 diabetes in FN adults. | NA | - Participants were concerned about loss of freedom, mortality, and the possibility of getting complications such as blindness, LEA, and kidney failure. |
| Brassard (1995) | 230 | P: -  R: James Bay Cree FN  O: -  G: 68 males; 162 females  R: -  E: -  S: -  S: -  Age (years) at diagnosis of diabetes: 48.3 SD 12.9 | Describe the epidemiology of the population with diabetes. | NA | - Macrovascular disease including ischemic heart disease, cerebrovascular disease and peripheral arterial disease: 14.4%   (Male 16.2%, Female 13.6%)   - Peripheral neuropathy: 9.6%   (Male:17.6%, Female 6.2%) |
| Macauley (1988) | 82 with  diabetes | P: South shore of the St. Lawrence River, R: Mohawk community of Kahnawake  O: -  G: 41 males; 59 females  R: -  E: -  S: -  S: 19 km from downtown Montreal; well organized patientcare, 92% of resident of Kahnawake are on the territory of a hospital  Age (years): 59 SD 10.9 | Define characteristics of diabetes related to vascular diseases. | Mohawk without diabetes  (n= 94) | - PAD (including ischemic foot, amputation and claudication) - n=12/82 compared to n= 2/94 adjusted OR 5.51 p= 0.038 (1.06-28.64)* - Patient had diabetes more than 10 years:   PAD = 25%   - Patient had diabetes 10 year or less:   PAD = 10%   - Neuropathy = 6% |
| Young (1985) | 385 | P: 30 isolated communities Northwestern ON and northeastern MB  R: Algonkian speaking  Cree and Ojibwa (Saulteaux)  O: -  G: 276 females; 109 males  R: -  E: -  S: -  S: -  Age (years):  0-14: 3  15-24: 12  25-44: 149  45-64: 165  65+: 56 | Describe epidemiologic data on diabetes. | Other Canadians | - Neuropathy = 5% |

**Legend: ***Statistically Significant **Abbreviations:** FN : First Nations; M: Métis; I: Inuit; DFU: Diabetic Foot Ulcers; LEA: Lower Extremity Amputation; N: Neuropathy; PAD: Peripheral Artery Disease; FD/C: Foot Deformities-Charcot; M: Mortality; DFI: Diabetic Foot Infection; QoL: Quality of Life; ORV: Other Relevant Diabetic Foot Variables; BKA: Below Knee Amputation
PROGRESS-Plus: P: Place of Residence; R: Race-ethnicity-culture-language; O: Occupation; G: Gender-sex; R: Religion; E: Education; S: Socioeconomic status; S: Social capital; A: Age; SD: Standard Deviation; NA: Not Available; NS: Not statistically significative
